# Altered plasma membrane abundance of the sulfatide-binding protein NF155 links glycosphingolipid imbalances to demyelination

**DOI:** 10.1073/pnas.2218823120

**Published:** 2023-03-30

**Authors:** Shannon J. McKie, Alex S. Nicholson, Emily Smith, Stuart Fawke, Eve R. Caroe, James C. Williamson, Benjamin G. Butt, Denisa Kolářová, Ondřej Peterka, Michal Holčapek, Paul J. Lehner, Stephen C. Graham, Janet E. Deane

**Affiliations:** ^a^Department of Clinical Neuroscience, Cambridge Institute for Medical Research, University of Cambridge, Cambridge CB2 0XY, UK; ^b^Cambridge Institute of Therapeutic Immunology and Infectious Disease, University of Cambridge, Cambridge CB2 0AW, UK; ^c^Department of Pathology, University of Cambridge, Cambridge CB2 1QP, UK; ^d^Department of Analytical Chemistry, University of Pardubice, Pardubice 53210, Czech Republic

**Keywords:** Krabbe disease, myelin, galactosylceramide, sulfatide, neurofascin

## Abstract

Myelin is a multilayered membrane that tightly wraps neuronal axons, enabling efficient, high-speed signal propagation. The axon and myelin sheath form tight contacts, mediated by specific plasma membrane proteins and lipids, and disruption of these contacts causes devastating demyelinating diseases. Using two cell-based models of demyelinating sphingolipidoses, we demonstrate that altered lipid metabolism changes the abundance of specific plasma membrane proteins. These altered membrane proteins have known roles in cell adhesion and signaling, with several implicated in neurological diseases. The cell surface abundance of the adhesion molecule neurofascin (NFASC), a protein critical for the maintenance of myelin-axon contacts, changes following disruption to sphingolipid metabolism. This provides a direct molecular link between altered lipid abundance and myelin stability. We show that the NFASC isoform NF155, but not NF186, interacts directly and specifically with the sphingolipid sulfatide via multiple binding sites and that this interaction requires the full-length extracellular domain of NF155. We demonstrate that NF155 adopts an S-shaped conformation and preferentially binds sulfatide-containing membranes in *cis*, with important implications for protein arrangement in the tight axon-myelin space. Our work links glycosphingolipid imbalances to disturbance of membrane protein abundance and demonstrates how this may be driven by direct protein–lipid interactions, providing a mechanistic framework to understand the pathogenesis of galactosphingolipidoses.

## Significance

Glycosphingolipids are an important class of lipids enriched in the outer leaflet of the plasma membrane. Disorders that alter glycosphingolipid metabolism cause devastating neurodegenerative and demyelinating diseases. We show that deletion of the glycosphingolipid metabolizing enzymes GALC or UGT8 cause significant changes to the abundance of specific plasma membrane proteins, several of which are implicated in degenerative brain disease. We extend this discovery by characterizing the specific interaction of the membrane protein neurofascin with the glycosphingolipid sulfatide. The extracellular domain of Neurofascin binds multiple sulfatide molecules, allowing it to bind flat along its own membrane, explaining its sensitivity to altered lipid composition. This work links diseases of glycosphingolipid metabolism to changes at the plasma membrane relevant to disease phenotypes.

Myelination of axons enables fast, saltatory impulse propagation, facilitating increased neural processing speed and energetic efficiency (1). Myelin is a multilayered membrane that is produced by oligodendrocytes in the central nervous system. Oligodendrocytes form numerous cellular extensions that wrap around axons to form distinct myelin internode segments. Contacts between the myelin and axonal membranes involve multiple protein–protein, protein–lipid, and possibly also lipid–lipid interactions (2–5). A key contact site, known as the paranode, is the region where the myelin sheath is anchored tightly to the axonal membrane, stabilized by the interaction between oligodendrocyte-specific and neurone-specific adhesion proteins (6). The paranode is also highly enriched in a specific class of lipids, the glycosphingolipids (GSLs), that partition into membrane microdomains in the outer leaflet of the plasma membrane and have numerous important roles in the function of the nervous system (7, 8). The importance of paranodal contacts is highlighted by the devastating demyelinating diseases that result from defects in the proteins that form these contacts or in changes to glycosphingolipid metabolism (9–14).

**Fig. 1.**
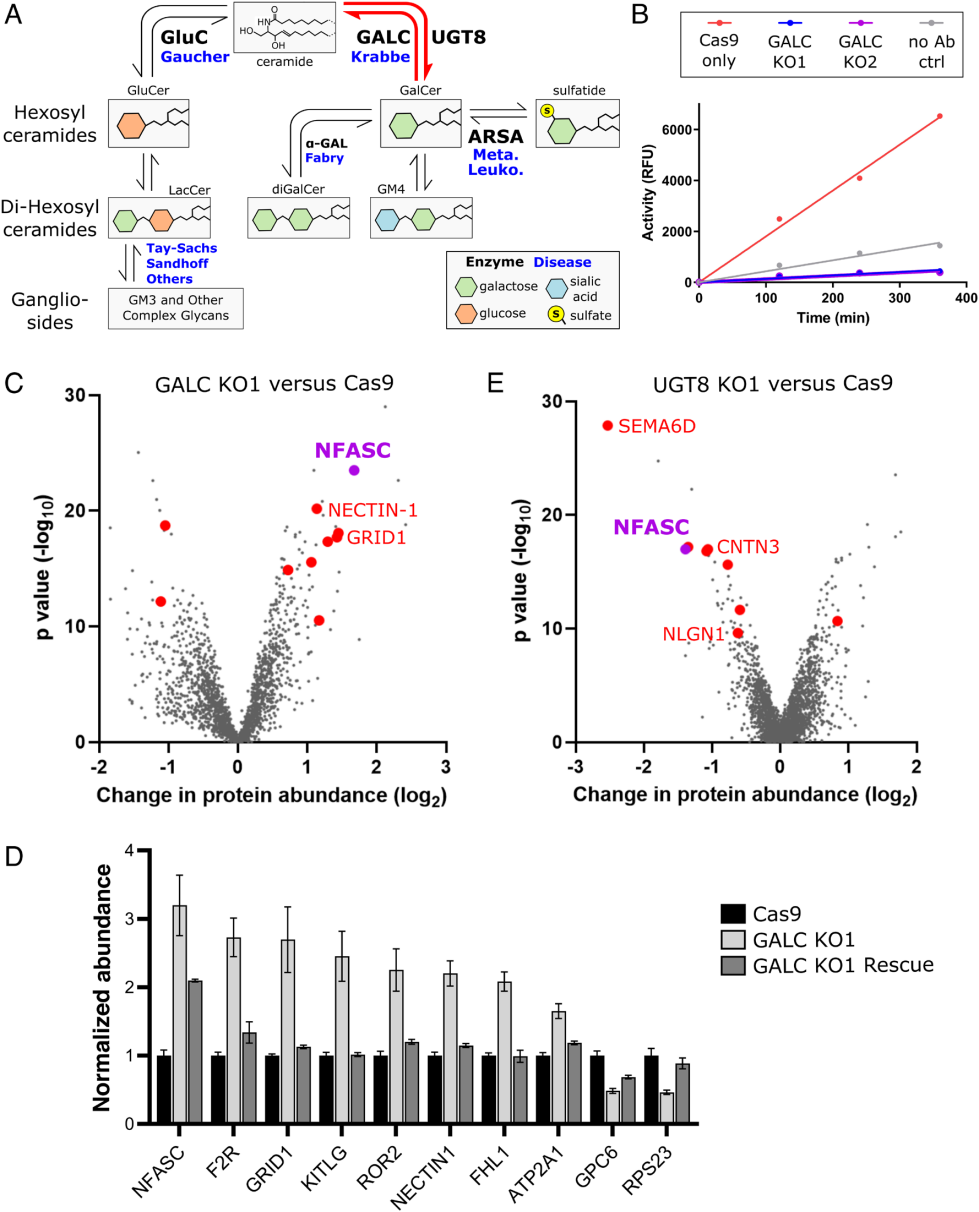
Disrupting galactosphingolipid metabolism alters the plasma membrane protein composition. (*A*) Simplified schematic diagram of GSL metabolism. Sequential addition or removal of glycans and sialic acid moieties produces a broad range of GSL headgroups. (*B*) GALC activity assays following immunoprecipitation (IP) from control and GALC knockout (KO) cell lines demonstrate loss of activity in the two clonal KO lines compared with Cas9-only control cells and a no-antibody (no Ab) control IP. Data are representative of n > 3 independent experiments. (*C*) Quantitative mass spectrometry following enrichment of plasma membrane proteins (PMP-MS) from a GALC KO1 cell line compared with the Cas9 control. A volcano plot with the horizontal axis showing average fold change across three biological replicates and the vertical axis showing significance (two-sided *t* test) across the three replicates. The 10 high-confidence targets (criteria as detailed in the main text) are colored in red with select targets labeled. (*D*) Normalized protein abundance values, from the PMP-MS data, for high-confidence targets in the Cas9 control, GALC KO1, and GALC rescue cell lines. Normalized abundances have been used to allow comparison across proteins with different absolute abundances in the cell (mean ± SE, n = 3). Additional PMP-MS supporting data are available in *SI Appendix*, Fig. S4*A* and Table S1. (*E*) As for panel (*C*) but for a UGT8 KO cell line. Additional PMP-MS supporting data are available in *SI Appendix*, Fig. S4*B* and Table S2.

The galactose-based GSLs, including galactosylceramide (GalCer) and its 3-*O*-sulfated derivative sulfatide, are highly enriched in the myelin membrane and are important for myelin integrity (15–17). The sphingolipidoses Krabbe disease and metachromatic leukodystrophy are fatal early-onset demyelinating diseases caused by defects in the enzymes β-galactosylceramidase (GALC) and arylsulfatase A (ARSA) that degrade GalCer and sulfatide, respectively (Fig. 1*A*) (18). These enzymes function in the lysosome to catalyze the stepwise removal of lipid headgroup moieties, facilitating the efficient recycling of these bioactive lipids. In disease, the lipid substrates and their deacylated derivatives, such as psychosine, accumulate and cause cell death. The majority of work to date has focused on the deleterious effects of lipid accumulation on lysosomal function and on the detergent-like properties of psychosine (19–21). Substantial evidence supports the “psychosine hypothesis” for Krabbe disease pathology, and blocking deacylation of GalCer to prevent psychosine production reduces Krabbe disease severity in mice (22). However, this work clearly demonstrated that psychosine is not the sole driver of disease and there is growing evidence in this and related sphingolipidoses that sphingolipid substrates accumulate throughout the cell, including at the plasma membrane (23–26). Sphingolipids also contribute to protein trafficking within the cell (27–29) and GSL imbalances change membrane protein abundance and function (26).

Mouse models of Krabbe disease and mice where GSL-synthetic enzymes have been ablated all display hypomyelination involving specific destabilization of contacts at the paranodal junction (16, 30–34). These models demonstrate that it is not just accumulation of GSLs, but also their absence, via loss of the UDP-galactosyl transferase 8 (UGT8 a.k.a. ceramide galactosyl-transferase) or ceramide sulfotransferase (CST) that can cause severe and rapid demyelination. As loss of GSL synthetic enzymes would not be expected to result in increased psychosine abundance, the molecular mechanisms underlying demyelination in these diseases may be more complex than psychosine-mediated cell death and involve specific changes at myelin-axon contact sites. We sought to determine whether the plasma membrane protein composition is changed in cell-based models of sphingolipid disorders and how these changes may contribute to disease phenotypes.

Here, we exploit oligodendrocyte-like cells to show that disruption of galactosphingolipid metabolism via deletion of the enzymes GALC and UGT8 specifically changes the plasma membrane proteome. The cell adhesion protein neurofascin (NFASC) is particularly sensitive to GSL imbalances, being reciprocally altered in its abundance at the PM when GSL synthesis and catabolism is inhibited. We demonstrate direct binding of the oligodendrocyte isoform of NFASC, NF155, to sulfatide but not to other closely related galactosphingolipids. Mapping of NF155 binding to sulfatide and structural characterization of its sulfatide-binding extracellular domain (ECD) shows how this essential component of the paranode may be oriented relative to the myelin membrane, enabling the tight membrane apposition required for paranode stability.

## Results

### Disrupting Galactosphingolipid Metabolism Alters the Plasma Membrane Protein Composition.

Krabbe disease pathology occurs in patients who possess <10% activity of the catalytic enzyme GALC. The majority of Krabbe disease patients possess a 30 kb deletion within this gene in at least one allele (21). Therefore, CRISPR/Cas9-mediated deletion of GALC represents a good model for this disease. Multiple independent clonal knockouts (KOs) of GALC were produced in the oligodendrocyte-like cell line, MO3.13 (35, 36). Genomic editing within early exons of the GALC gene was verified by sequencing across the guide RNA (gRNA)-target sites (*SI Appendix*, Fig. S1 *A* and *B*). To test for GALC enzyme activity in these cells, a highly sensitive activity assay was performed following enrichment of any residual GALC protein via immunoprecipitation (IP) (Fig. 1*B*). These data confirm loss of GALC activity in two independent GALC KO cell lines generated using gRNAs targeting different regions of the gene. To facilitate comparative analysis of how galactose-based GSLs influence cell function, additional cell lines were generated knocking out the synthetic enzyme UGT8. This enzyme functions in the endoplasmic reticulum to add the galactose headgroup to ceramide, forming the backbone of all galactosylated-sphingolipids (Fig. 1*A*). Gene editing was verified by sequencing across the gRNA-target sites (*SI Appendix*, Fig. S1 *C* and *D*). Mass spectrometry-based lipidomic analysis specifically tailored for detection of glycosylated sphingolipids demonstrated accumulation of several GSL species in the GALC KO cells and reduced abundance of GSLs in the UGT8 KO cells (*SI Appendix*, Fig. S2).

We quantified the differential expression of plasma membrane proteins between wild-type (WT) cells, Cas9-only control cells, the two independent GALC KO clones generated using different gRNAs, and a GALC rescue line where GALC expression had been restored (*SI Appendix*, Fig. S3). Intact cell monolayers were labeled with activated aminoxybiotin to allow enrichment of surface proteins via streptavidin-affinity prior to mass spectrometry analysis (37). Plasma membrane profiling mass spectrometry (PMP-MS) data were collected for 3,460 proteins quantified in all datasets, allowing several criteria to be used for determining confidence in the target identification. Protein abundance changes in the two independent GALC KO cell lines were compared with Cas9 controls (Fig. 1*C* and *SI Appendix*, Fig. S4*A*), and high-confidence targets were selected based on: no significant change between WT and Cas9-only control lines; significant change in membrane abundance in both KO lines; change in abundance by more than twofold in at least one KO line; identification by two or more unique peptides; and significant recovery following reexpression of GALC (Fig. 1*D*). These stringent criteria identified 10 high-confidence hits, eight of which showed increased abundance at the cell surface of KO cells (*SI Appendix*, Table S1). These proteins include NECTIN-1, NFASC, and GRID1 (a.k.a. GluD1) with important roles in cell adhesion, axon-glial contacts, synapse formation, and neurotransmission (38–40), highlighting their potential roles in the demyelinating neurodegenerative pathologies seen in Krabbe disease. As only a small subset of PM proteins were altered in these cell lines, the observed changes are likely to be caused by direct effects rather than by nonspecific fluctuations in membrane fluidity that would be expected to influence the abundance of many PM proteins indirectly.

To probe which of the GALC high-confidence hits may be directly sensitive to changes in GSL abundance, as opposed to downstream cellular consequences of these changes, PMP-MS analysis was also performed on the UGT8 KO cell lines and compared to Cas9 control cells, applying equivalent criteria as above (Fig. 1*E* and *SI Appendix*, Fig. S4*B*). Nine high-confidence hits were significantly altered in both UGT8 KO cell lines (*SI Appendix*, Table S2). All but one of these proteins has lower abundance at the PM versus the control cells, in a pattern opposite to that seen for the GALC KO cells. The high-confidence hits again include several proteins, such as CNTN3, NLGN1, and SEMA6D with important roles in cell adhesion and neuronal function (41–43). Interestingly, NFASC (neurofascin) was identified again as a high-confidence target with reciprocal changes in cell surface abundance, being increased in GALC KO cells and decreased in UGT8 KOs (Fig. 1 *C* and *E*). NFASC is an L1-type cell adhesion protein from the immunoglobulin superfamily, with crucial roles in neuronal development and myelination (39). Loss of a specific isoform of NFASC (NF155) leads to disorganization of the paranode (11) and causes a severe neurodevelopmental disorder (44). NF155 is part of a multiprotein complex at the paranode that bridges the myelin-axon contact site (45, 46), and the stability of this complex depends on the presence of GSLs (3, 47). Interestingly, the PM proteomic data determined here for both GALC and UGT8 KOs identify that NFASC abundance at the cell surface is sensitive to, and correlated with, GSL abundance.

### The NFASC Isoform NF155 Specifically and Directly Binds Sulfatide.

**Fig. 2.**
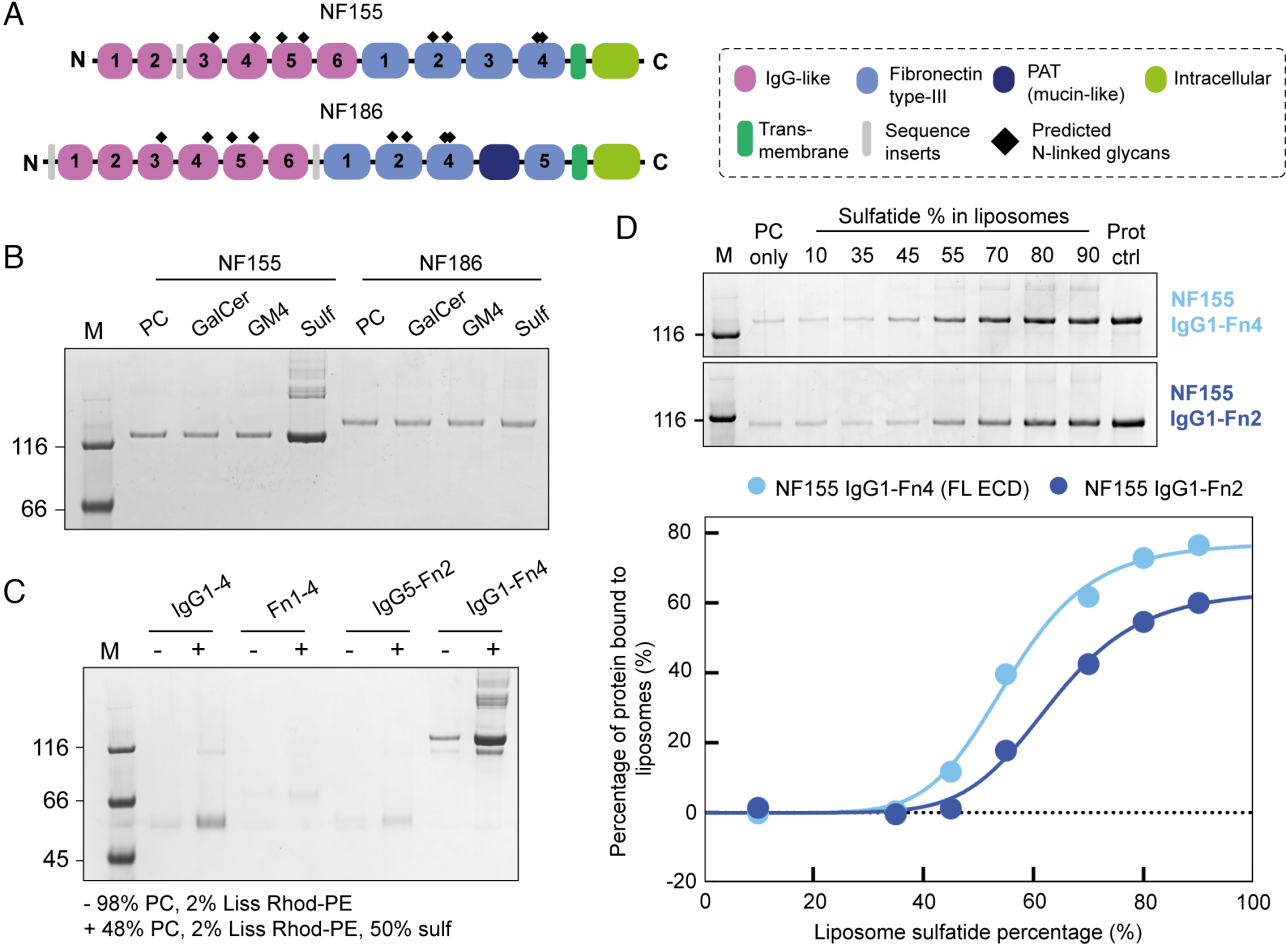
Lipid-binding assays demonstrate that NF155 specifically and directly binds sulfatide. (*A*) Protein domain schematic of NF155 and NF186, highlighting the differences between the two, arising from alternative splicing. The IgG-like domains are in pink, the Fn3-like domains are in light blue, the PAT domain is in dark blue, the transmembrane domain is in dark green, the intracellular domain is in light green, and the predicted N-glycosylation sites are highlighted by black diamonds. (*B*) Liposome-binding assay demonstrating that NF155-ECD binds specifically to liposomes containing sulfatide, and not to the other closely related GSLs, GalCer, and GM4, while NF186 does not bind (above background). Assays were performed with 1 µM protein and 0.8 mM liposomes. PC liposomes contain 98% PC and 2% Rhod-PE. GalCer, GM4, and Sulf liposomes contain 48% PC, 2% Rhod-PE, and 50% of either GalCer, GM4 or sulfatide, respectively. (*C*) Mapping the sulfatide-binding region using NF155-ECD truncations (1 µM protein, 0.8 mM liposomes) demonstrates that only the full-length (FL) ECD shows significant binding to liposomes containing 50% sulfatide. (*D*) *Top*: Liposome-binding assay performed with 250 nM NF155 using 1.6 mM liposomes containing increasing concentrations of sulfatide (IgG1-Fn4, top gel, IgG1-Fn2, lower gel). *Bottom*: Densitometric analysis of the SDS PAGE reveals a sigmoidal binding relationship for both NF155 constructs (IgG1-Fn4, light blue, IgG1-Fn2, dark blue). The quantification is calculated from the gels shown above and is representative of four independent experiments. Prot ctrl represents total protein added to each assay sample. Replicate data are provided in *SI Appendix*, Fig. S8. A total of n = 4 independent experiments were performed.

NFASC possesses multiple splice variants, each with distinct functions, localization, and expression patterns (48). To probe the mechanism underlying NFASC abundance changes in response to sphingolipid imbalances, we focused on the two most well-studied isoforms expressed in the CNS, NF155, and NF186 (Fig. 2*A*). NF155 is expressed specifically in oligodendrocytes and localizes to the paranode, where it forms a tight septate-like junction between the myelin and axonal membranes through interaction with the axonal proteins contactin 1 (CNTN1) and contactin-associated protein (Caspr) (9, 45). This junction is crucial as a diffusion barrier between the voltage-gated sodium channels (NaVs) in the node and voltage-gated potassium channels in the juxtaparanode. In contrast, NF186 is expressed in neurones and localizes to the nodes of Ranvier, where it clusters NaVs and interacts with components of the extracellular matrix, contributing to the node of Ranvier assembly (49). Therefore, both isoforms play key but distinct roles in promoting efficient signal conduction along the neuronal axon. These two isoforms differ in domain organization, particularly at the C-terminal end of the ECD where NF155 has four fibronectin type-III (Fn) domains (Fn1-4), while NF186 has Fn domains 1, 2, 4, and 5 with a PAT (Proline/Alanine/Threonine, mucin-like) domain between Fn4 and 5 (Fig. 2*A* and *SI Appendix*, Fig. S5). To test whether NFASC isoforms bind GSLs directly, and to probe their binding specificity, the full-length (FL) ECDs of NF155 and NF186 were expressed in the human cell line HEK293-F, secreted into the medium, and purified maintaining native glycans for testing in liposome interaction assays. Three galactosylated sphingolipids, GalCer, sulfatide, and GM4, were individually incorporated into liposomes containing phosphotidylcholine (PC) and rhodamine B-labeled phosphotidylethylolamine (Rhod-PE) and tested for binding to FL NF155-ECD and NF186-ECD (Fig. 2*B*). Both NF155 and NF186 possess some binding to PC-containing liposomes. However, despite NF186 and NF155 being closely related isoforms, only NF155 displayed dramatically increased binding to liposomes containing sulfatide with no increased binding to liposomes containing the closely related lipids GalCer or GM4. To ensure that this binding was specific for sulfatide and not mediated by nonspecific electrostatic interactions, an alternative anionic lipid, phosphatidylserine (PS) was tested in liposome-binding assays with no detectable binding (*SI Appendix*, Fig. S6).

To explore the potential effects of NF155 glycosylation on sulfatide binding, we produced NF155-ECD in the presence of kifunensine, to yield protein with high-mannose glycosylation, which can be removed with endoglycosidase H (Endo H). The high-mannose and deglycosylated forms of NF155-ECD showed the same binding to sulfatide-containing liposomes as the standard complex human glycosylated form (*SI Appendix*, Fig. S7), supporting that the interaction with sulfatide is mediated by direct binding to the protein and is not a glycan–glycan interaction.

To map which domain(s) mediate sulfatide binding, NF155 truncations were expressed and purified encompassing regions that differ between NF155 and NF186 isoforms: the four N-terminal Ig domains (IgG1-4); the four C-terminal Fn domains (Fn1-4); and a central region (IgG5-Fn2). Liposomes containing 50% sulfatide were used for interaction assays, revealing that IgG1-4 retains some sulfatide binding, while Fn1-4 and IgG5-Fn2 possess barely detectable binding to liposomes (Fig. 2*C*). Interestingly, the FL NF155-ECD shows much stronger binding to sulfatide-containing liposomes than any truncation, suggesting avidity-enhanced binding to multiple, distinct, and weak sulfatide-binding sites. To assess potential binding cooperativity, binding assays were performed using 1.6 mM liposomes containing increasing concentrations of sulfatide (0 to 90%) and incubated with 250 nM NF155-ECD (Fig. 2*D* and *SI Appendix*, Fig. S8, *light blue*). Densitometric quantification of NF155 bands following SDS-PAGE identified a sigmoidal relationship between NF155 binding and the sulfatide content of liposomes (Fig. 2*D*). Fitting the data to the Hill equation gives a Hill coefficient (7.7 ± 0.9, mean ± SE) consistent with NF155 possessing multiple sulfatide-binding sites and that the binding of ligands is cooperative.

Domain mapping experiments suggested that the membrane-proximal C-terminal Fn domains are dispensable for the interaction with sulfatide (Fig. 2*C*). Therefore, a construct of the NF155-ECD missing Fn3-4 (IgG1-Fn2) was purified and assayed for binding to sulfatide-containing liposomes (0 to 90%) (Fig. 2*D* and *SI Appendix*, Fig. S8, *dark blue*). Surprisingly, IgG1-Fn2 showed a reduction in sulfatide binding when compared with the FL ECD, which is not caused by protein aggregation or oligomerization as a consequence of the truncation as shown by multiangle light scattering coupled to size-exclusion chromatography (SEC-MALS) analysis (*SI Appendix*, Fig. S9). The fraction of protein that binds to liposomes is significantly decreased (IgG1-Fn2: 55.0% ± 7.0%, IgG1-Fn4: 76.8% ± 2.1% at saturating sulfatide concentrations; mean ± SE, n = 4, paired *t* test *P* = 0.025), and the percentage sulfatide required for half-maximal binding was significantly increased (IgG1-Fn2: 62.3% ± 2.1%, IgG1-Fn4: 55.8% ± 1.4%; *P* = 0.02) suggesting increased membrane dissociation for the truncation. However, the Hill coefficient was not significantly different (IgG1-Fn2: 8.4 ± 0.7; IgG1-Fn4: 7.7 ± 0.9; *P* = 0.3). This suggests that removal of the last two Fn domains did not remove a sulfatide-binding site, but the presence of these domains increases the affinity of NF155 for sulfatide. The conformation of the FL ECD may therefore be important for efficient membrane association.

### NF155-ECD Adopts an S-Shape.

**Fig. 3.**
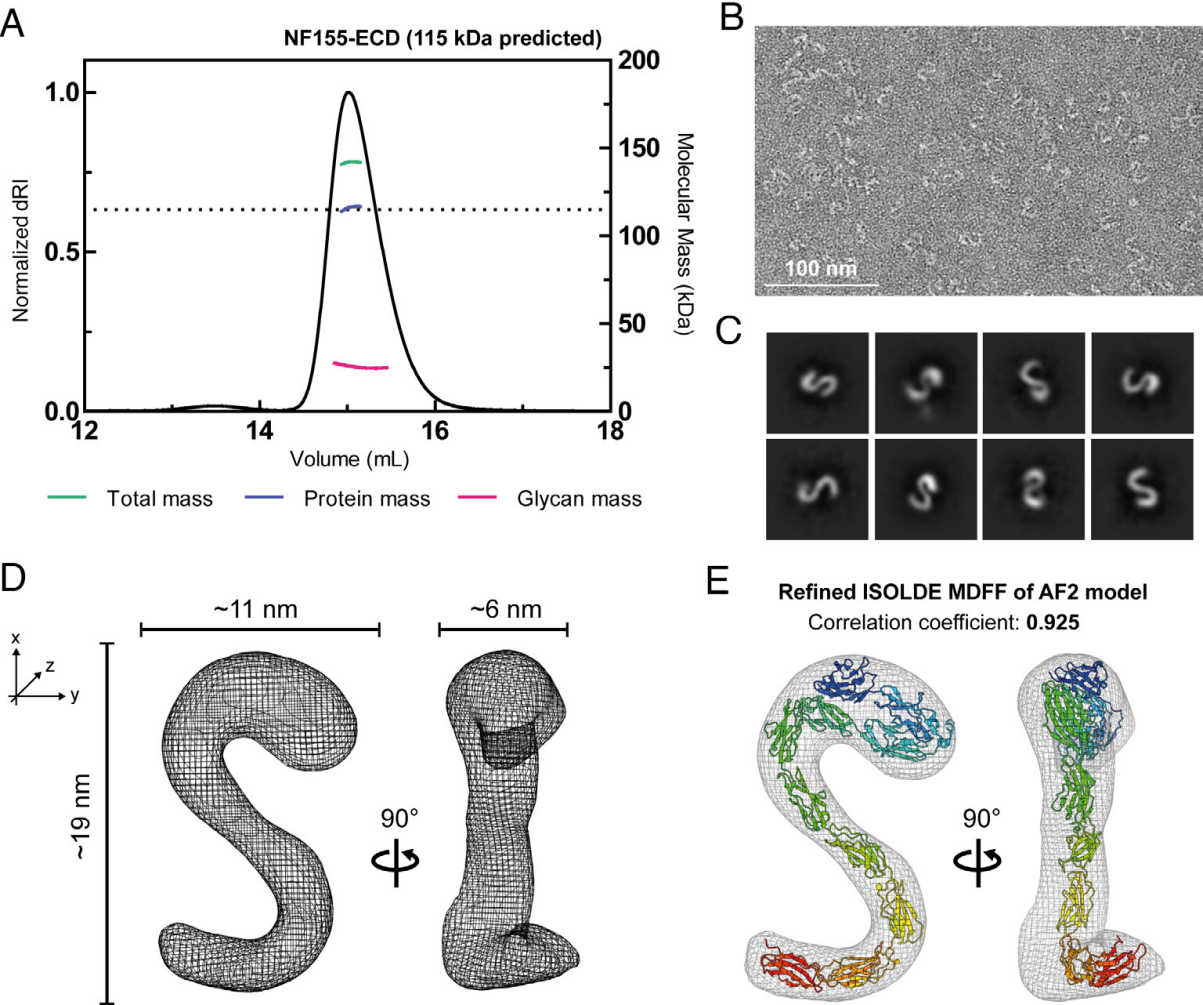
The extracellular domain (ECD) of NF155 is monomeric and adopts an “S”-shape. (*A*) Size exclusion chromatography coupled to multiangle light scattering (SEC-MALS) demonstrates that the NF155 FL ECD is monomeric with a protein mass of 115 kDa (consistent with the predicted mass, dotted line) and 25 kDa glycans. (*B*) Raw image of NF155 FL ECD single particles, stained with 2% uranyl acetate. (*C*) Selection of 2D class averages generated from the negative-stain EM data using CryoSPARC. (*D*) The 3D reconstruction of the NF155 FL ECD, generated using CryoSPARC analysis of the negative stain EM data. The particle possesses dimensions 19 × 11 × 6 nm, two orientations are shown rotated by 90°. (*E*) The best AF2-generated model was fit to the EM map using ISOLDE molecular dynamics flexible fitting (MDFF), resulting in a high-quality fit (correlation coefficient = 0.925).

To date, the only experimental structural data available for NFASC is a crystal structure of the four N-terminal Ig domains (50) that fold back upon themselves to form a globular “horseshoe” arrangement. This work also suggested that NFASC may form homodimers (50). To explore how the conformation of the FL NF155-ECD may contribute to its membrane-binding behavior, we employed a range of biophysical and structural techniques. Using SEC-MALS, we demonstrated that the FL, glycosylated NF155-ECD is a monomer (Fig. 3*A*). This difference with the published literature may be attributed to previous studies using truncated NFASC constructs or the removal of surface glycans, as we observed that deglycosylation of NF155 destabilizes the protein resulting in time-dependent aggregation and degradation. As our data have implicated that the FL ECD is required for binding, subsequent structural studies were carried out using the fully glycosylated FL ECD. Attempts to crystallize this construct were unsuccessful, potentially due to the heterogeneity of the glycans. Recent advances in artificial intelligence-based deep learning strategies, exemplified by AlphaFold2 (AF2), allow for the highly accurate prediction of protein structures (51). Structural predictions of NF155 using ColabFold (52), implementing AF2, provide accurate models for the individual domains of NFASC. However, the overall conformation and relative interdomain orientations [described by the Predicted Alignment Error (PAE) plot] are poorly predicted outside the IgG1-4 region, limiting our understanding of the overall ECD shape (*SI Appendix*, Fig. S10). The NF155-ECD is near the lower size limit for cryo-electron microscopy (EM) structure determination, and preliminary data collection did not yield high-quality particle analysis. We therefore used negative-stain EM to capture images of the overall ECD structure. Raw images of the NF155 FL ECD sample reveal clear, high-contrast particles that appear monodisperse and homogenous (Fig. 3*B*). In total, 21,703 particles were analyzed, allowing class averaging (Fig. 3*C*) and single-particle reconstruction (Fig. 3*D*). This three-dimensional (3D) reconstruction, at 19.2 Å (*SI Appendix*, Fig. S11), reveals that the NF155-ECD possesses an asymmetric S-shaped conformation with a bulbous “head” followed by two sharp bends (Fig. 3*D*). This structure also shows that, although the first four IgG domains are known to fold back upon themselves, the full ECD is not globular, adopting a relatively flat shape with dimensions 19 × 11 × 6 nm (Fig. 3*D*).

Initial rigid-body docking of the AF2 models for the FL NF155-ECD did not fit the EM density maps well (*SI Appendix*, Fig. S12*A*), with modest correlation coefficients (CC = 0.748 to 0.859). Flexible fitting of the best AF2 model (model rank 1) into the EM map using molecular dynamics simulations as implemented by ISOLDE (53) in ChimeraX (54) allowed for a better fit (CC = 0.913, *SI Appendix*, Fig. S12*B*). However, visual inspection of the fitted structure into the EM map suggested that the “head” domain encompassing IgG1-4 was not oriented optimally to explain the density. Therefore, the IgG1-4 domain was manually rotated as a rigid body, maintaining connectivity to IgG5. Following molecular dynamics-based flexible fitting, as above, the final model displayed an excellent fit to the map (CC = 0.925, Fig. 3*E*).

### NF155 Membrane-Binding Orientation.

**Fig. 4.**
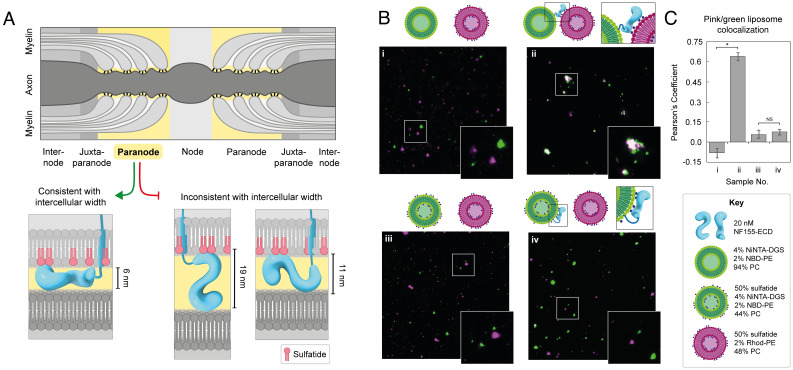
NF155 membrane orientation. (*A*) The paranodal junction (yellow) is estimated to possess an intermembrane gap of 3 to 8 nm, a width that is only consistent with a model of NF155 FL ECD lying flat along the myelin membrane, potentially stabilized by its interaction with multiple sulfatide headgroups (red). (*B*) Liposome-binding assays performed at 20 nM NF155 with 0.4 mM liposomes, demonstrating clustering of NBD-labeled liposomes containing 1% NiNTA-DGS (green) with rhodamine-labeled liposomes containing 50% sulfatide (pink) in the presence of the NF155-ECD (sample *ii*). This clustering is prevented when NF155 is preincubated with NBD-labeled liposomes containing 50% sulfatide (sample *iv*). No clustering is seen when the liposomes are incubated in the absence of NF155-ECD (samples *i* and *iii*). Images shown here represent 8% of the full image area. (*C*) Quantification of the colocalization of the pink and green liposomes in the samples described in (*B*). Mean ± SD is shown for quantification from three full images. Significance calculated using a two-tailed unpaired *t* test, *P ≤ 0.0001, NS = not significant.

NF155 is localized to the paranode, a septate-like junction between myelin and axonal membranes that are separated by a gap of around 3 to 8 nm (55, 56). The dimensions of the NF155-ECD structure are 19 × 11 × 6 nm (Fig. 3*C*), suggesting that in order to fit within this tight intermembrane space, NF155 must lie flat (Fig. 4*A*). As both NF155 and sulfatide reside in the membrane of myelin-forming cells, the sulfatide-binding data suggest that NF155 binds in *cis* to the membrane within which it is embedded. To test this hypothesis, a liposome clustering assay was developed incorporating different fluorescent lipids to facilitate direct visualization of the interaction. At high concentrations, NF155 caused liposomes to coalesce forming large assemblies that compromise visualization (*SI Appendix*, Fig. S13*A*). Therefore, these assays employed a relatively low concentration of NF155 (20 nM). For initial clustering assays, one population of liposomes was produced incorporating Rhod-PE (pink) and sulfatide, while a second population contained NBD-PE (green) and Ni-NTA conjugated lipids (NiNTA-DGS). Following mixing, liposomes were inspected by wide-field microscopy and did not show clustering of different colored liposome populations (Fig. 4 *B*, *i*). Upon addition of NF155-ECD, which can be incorporated via its C-terminal His_6_ tag into the green Ni-containing liposomes, significant clustering of pink and green liposomes is observed (Fig. 4 *B*, *ii* and *C*). In this case, NF155-ECD clusters the different liposome populations by being anchored into the green Ni-containing liposomes and binding to the sulfatide present in the pink liposomes (Fig. 4*B*, schematic). Additional clustering assays were carried out to test whether incorporation of sulfatide into both liposomes could inhibit clustering (Fig. 4 *B*, *iii* and *iv*). Incorporation of sulfatide into both liposomes did inhibit clustering in the presence of NF155-ECD, suggesting that NF155 can bind to the membrane within which it is embedded (binding in cis) and that this buries the sulfatide-binding sites. Taken together, these data reveal an intriguing membrane association model for NF155, in which the ECD has an extended sulfatide-dependent interaction with the membrane it is embedded in, maintaining it in a “flat” conformation relative to the membrane that is consistent with the size of the intermembrane space at the paranode.

## Discussion

Here, we have demonstrated that disruption of GSL metabolism in oligodendrocyte-like cells alters the abundance of PM proteins. Loss of the GSL metabolic enzymes GALC and UGT8, which remove or add a galactose headgroup from/to ceramide, respectively, results in significant changes in the abundance of specific plasma membrane proteins. Importantly, the small subset of altered proteins suggests that these changes are direct effects mediated by changes in the abundance of specific lipids arising from the loss of these enzymes, rather than nonspecific modulation to membrane fluidity. Of particular interest is the observation that NFASC shows reciprocal changes in abundance that correlate with GSL abundance. This finding suggested that a direct NFASC–GSL interaction was driving the change in NFASC abundance at the PM. We directly demonstrate in vitro that the NF155 isoform of NFASC interacts specifically with sulfatide but not with other closely related or similarly charged lipid species, while the NF186 isoform does not. Despite being extremely similar (Fig. 2*A* and *SI Appendix*, Fig. S5), these two isoforms have different roles within the nervous system that, as demonstrated by our data, are likely driven by distinct interactions with other cellular components. Direct binding of the NF155-ECD to sulfatide demonstrated here provides a molecular explanation for previous observations that NF155, but not NF186, is resistant to detergent solubilization, suggesting that it associates with lipid rafts in vivo (57) and that this requires sulfatide (58). The absence of lipid raft-associated NF155 in UGT8 KO mice explains the disruption of the paranodal junction (57). This study did not identify a reduction in NF155 abundance in whole-brain homogenates or cultured oligodendrocytes (57), in contrast to our PMP-MS data where cell-surface NF155 abundance was altered. A possible explanation for this could be that disrupted partitioning of NF155 into lipid rafts arising from GSL imbalances could alter NF155 abundance at the PM specifically, while its whole-cell abundance may remain unchanged.

The PMP-MS data for the GALC rescue cell line provide strong evidence for the direct importance of GALC in maintaining PM homeostasis. The restoration of protein abundances back to normal levels was excellent for the 10 high-confidence targets (Fig. 1*D*), confirming that these were direct effects of the loss of GALC and not off-target effects. Despite significant recovery, the abundance of NFASC at the PM recovered less well than other targets. Re-expression of GALC was induced by addition of doxycycline prior to differentiation for 7 d, and this may be insufficient to restore key GSL species to normal levels. The direct binding of NFASC to sulfatide may make it especially sensitive to cellular lipid composition, requiring a longer rescue of GALC expression for full recovery. The data presented here demonstrating reciprocal changes in NFASC abundance at the PM in cells accumulating (GALC KO) or deficient (UGT8 KO) in GSLs, along with the demonstration of direct binding of NF155 to sulfatide, suggest several potential mechanisms for how NFASC accumulates in membranes. Direct binding supports the hypothesis that NFASC and sulfatide traffic together through the secretory and endosomal pathways, resulting in a change in abundance for both in different cellular compartments, including the PM. Direct binding may alter NF155 stability and retention at the PM, or could influence its proteolytic shedding (59). However, the precise mechanism of NFASC accumulation at the PM in response to GSL changes remains unclear.

Liposome-binding assays were conducted using truncations of NF155 to map the specific sulfatide-binding domain(s) (60). Both N- and C-terminal truncations significantly reduced binding to sulfatide-rich membranes, and the binding of the FL NF155-ECD revealed that it possesses multiple sulfatide-binding sites that display positive cooperativity. Due to the relatively small size of the sulfatide headgroup, a single galactose with one sulfate group, this avidity effect would help facilitate high affinity binding to sulfatide-containing membranes. Sulfatide is highly enriched at myelin-axon contact sites, as demonstrated by antisulfatide antibody binding to myelinated axons (61). This enrichment may result in extremely high local concentrations of sulfatide, helping facilitate multivalent interactions. Interestingly, our data do not support a simple model where NF155 comprises a linear arrangement of tandem Ig and Fn domains extending away from the PM. Instead, for NF155 to engage with several sulfatide headgroups along its length, it must either fold back toward or lie flat along the membrane. Our structural analysis shows that the FL NF155-ECD adopts an S-shaped conformation, bending back upon itself several times, with a dense N-terminal “head” domain and a highly curved C-terminal region. Previous work has demonstrated that loss of NF155 Ig domains 5 and 6 abolished the formation of paranodal junctions (60). The structural data presented here suggest that loss of these domains would have a substantial impact on the overall conformation of NF155, which could have severely impaired sulfatide binding and interrupted its ability to bind the paranodal proteins CNTN1 and Caspr.

Bent or curved structural conformations, such as seen here for the NF155-ECD, have been observed previously for other proteins involved in cell–cell contacts such as contactins and sidekick proteins, where their shape was proposed to facilitate both cis and trans interactions with membranes or other membrane proteins (62, 63). In these cases, this arrangement was inferred from structures of fragments of these proteins rather than FL ECD conformations. Importantly, the dimensions of the NF155-ECD structure combined with liposome clustering assays support that the ECD of NF155 lies flat within the gap at the paranode, contacting the myelin membrane via multiple sulfatide-binding sites. Although not explored in detail here, it is clear that the complex formed with CNTN1 and Caspr must fit within this tight intercellular space, suggesting that these proteins must also adopt relatively flat conformations. Structural predictions of CNTN1 and Caspr ECDs possess dimensions that would be compatible with fitting in the paranodal gap (*SI Appendix*, Fig. S14). Recently, a small-angle X-ray scattering structure of the FL CNTN1 ECD demonstrated that in solution this protein is monomeric, elongated, and flexible (64). This structure is distinct from the AF2 predictions, highlighting the importance of determining experimental structures of these paranodal proteins in complex with each other to understand how they assemble together to form and stabilize the paranode. In a CST KO mouse model, loss of NF155 at the paranode was age dependent, suggesting that sulfatide is not necessary for initial formation of the paranodal junction but very important for its long-term stability. Based on our data, this stability may be driven by the multiple sulfatide-binding sites in NF155 forming an extensive interaction with the membrane that could promote tightening of the paranodal gap and facilitate interactions with axonal proteins, CNTN1 and Caspr, by displaying the correct interaction interfaces. The extensive protein–protein and protein–lipid interactions that occur at the paranode are likely to function together to support the long-term stability of the paranode.

Previous work using mouse models where galactose-based sphingolipids are dysregulated by either accumulation due to GALC loss (Twitcher mouse), or deficiency due to UGT8 or CST loss, all manifest with defective myelination (16, 31, 34). Interestingly, this means that rather than reciprocal changes in lipid abundance leading to reciprocal changes in myelin integrity, it is the precise homeostasis of GSL levels that is essential for stable myelin formation. Lipidomics data from GALC and UGT8 KO cell lines suggest that disruption to GSL metabolism results in complex lipid changes, including altered abundance of several GSL species (*SI Appendix*, Fig. S2). This is consistent with previous studies showing unexpected disruption to sphingomyelin and phospholipid abundances in CST KO mice (65), supporting that it is not just the direct enzyme substrates that accumulate in GSL diseases. This complexity makes it challenging to determine which lipid species may be the primary driver of myelination defects and it seems likely that a combination of changes to multiple lipid species and membrane proteins drives severe disease. Although sulfatides were not able to be quantified in these cell lines, the changes to NFASC abundance are consistent with changes seen in models and diseases of sulfatide imbalance. Specifically, CST KO mice display node/paranode disorganization and suffer from age-dependent neurodegeneration due to loss of paranodal NF155, while NF186 localization to the node is unchanged (3). Similarly, patients with metachromatic leukodystrophy are unable to break down sulfatide due to the loss of ARSA, leading to sulfatide accumulation in both the lysosome and myelin membrane, causing rapid demyelination (26). These data support that both accumulation and absence of sulfatide causes myelination defects, and that NF155 and sulfatide may play unexpected but important roles in the pathology of Krabbe disease. Interestingly, disruption of GALC gene expression and enzyme activity in neuronal cells does not result in any significant changes to the abundance of PM proteins (*SI Appendix*, Fig. S15). These data support the hypothesis that altered GSL levels will have the greatest impact in the cell type where the lipid is most highly abundant. However, it is possible that cellular components, including lipids, are exchanged between oligodendrocytes and axons via exosomes (endosome-derived microvesicles) (66). The importance of axon–glial interactions and the potential exchange of bioactive molecules between these cell types may contribute to the axonal pathologies identified in Krabbe disease models (67).

In addition to genetic demyelinating diseases, such as Krabbe and metachromatic leukodystrophy, antibodies against paranodal components are present in several autoimmune mediated demyelinating diseases (68, 69). Autoantibodies against NF155 and sulfatide have been identified in chronic demyelinating conditions, such as multiple sclerosis (70, 71) and chronic inflammatory demyelinating polyradiculoneuropathy (14, 72), as well as acute demyelinating conditions such as Guillain–Barré syndrome (73, 74). The presence of anti-NF155 antibodies is often associated with a more severe clinical course and worse prognosis (73, 75). Although the exact mechanisms remain unclear, these antibodies may directly interfere with protein–protein and protein–lipid interactions to disrupt axon-myelin contacts and drive antibody-mediated demyelination (76).

Understanding how loss of NF155, a crucial junctional protein, would destabilize axon-myelin contacts is clear, but it is less obvious how increased abundance might also destabilize these contacts. It is possible that accumulation of NF155 at the PM of myelin-forming cells might interfere with the correct formation of the axon-myelin protein complexes, specifically the interactions between NF155 and the axonal proteins CNTN1 and Caspr. Alternatively, the high abundance of NF155 combined with its ability to interact with membranes may have a destabilizing effect on the membrane similar to that caused by membrane-embedding antimicrobial peptides (77). In support of this, we observed that at high concentrations, NF155 can induce membrane destabilization and blebbing of liposomes and cells in vitro (*SI Appendix*, Fig. S13*B*). However, the relevance of this to demyelinating processes in vivo remains unclear. Finally, there is evidence that during myelin compaction there must be reduction in the charged components of the glycocalyx, including sialic acid residues, in order to avoid electrostatic repulsion between membranes (78). It is therefore possible that, in addition to a specific role for NF155 in Krabbe disease, increased abundance of other lipids and proteins, as seen in the GALC KO, may hinder myelination via increased electrostatic repulsion.

This work demonstrates the exquisite sensitivity of the PM proteome to alterations in lipid homeostasis. Several PM proteins with important roles in cell adhesion, neurotransmission, and synapse formation were altered in this cell-based model of Krabbe disease, highlighting potential leads in understanding the molecular consequences of GSL accumulation. In particular, the critical paranodal complex protein NF155 exhibits specific and highly cooperative binding to the GSL sulfatide. Taken together, our results suggest that the flat and highly curved NF155-ECD interacts extensively with the myelin membrane in which it is embedded, providing important insights into the potential arrangement of paranodal proteins within the tight intermembrane space at the paranode. Furthermore, our work highlights how pathology in sphingolipidoses may be driven by disruption of specific and distinct GSL–protein interactions.

## Methods

### Cells and Cell Culture.

Human oligodendrocyte-like MO3.13 cells were a gift from Louise Boyle (University of Cambridge). For normal maintenance, cells were cultured in Dulbecco's Modified Eagle Medium (DMEM) containing 4,500 mg/l-glucose, l-glutamine, sodium pyruvate, and sodium bicarbonate supplemented with 10% (v/v) fetal calf serum (FCS). Differentiation was induced by culturing in DMEM, 1% (v/v) FCS, and 100 nM phorbol 12-myristate-13-acetate for 7 d with fresh medium applied every 2 to 3 d. The cells were cultured at 37 °C in a humidified 5% CO_2_ atmosphere. Details of neuronal cell line culture and KO generation are provided in *SI Appendix, Methods*.

### CRISPR/Cas9 Genome Editing.

Oligonucleotides encoding single-guide RNAs (*SI Appendix*, Table S3) targeting human GALC exon 3 and UGT8 exon 2 were cloned into the *Bbs*I restriction site in pSpCas9(BB)-2A-Puro as previously described (79). MO3.13 cells were transfected with 2 µg of each plasmid (including an untargeted Cas9 control) using FuGeneHD transfection reagent as per manufacturer’s instructions. Following overnight incubation, selection with 2 µg/mL puromycin was applied for 2 d. Clonal cell lines were established by limiting dilution. After expansion of clones, editing at the relevant genomic site was confirmed by amplification and sequencing of PCR product from genomic DNA (*SI Appendix*, Table S4) and analyzed using TIDE (80).

### Generation of GALC Rescue Cell Line.

Human GALC cDNA was cloned into the lentiviral vector pCW57 using restriction sites *Nhe*I and *Sal*I. For lentiviral production, 6 µg pCW57-hGALC was cotransfected into 1.2 × 10^7^ HEK293-T cells with 12 µg psPAX2 and 3 µg pMD2.G viral packaging and envelope vectors (gifts from Hayley Sharpe, Babraham Institute, Cambridge) using FuGeneHD transfection reagent. After 8 h, the transfection medium was replaced with fresh DMEM containing 10% (v/v) FCS and incubated for 48 h. Virus-containing supernatant was concentrated 200-fold by ultracentrifugation (100,000 g, 1.5 h) and 25 µL was added dropwise to a 6-well plate containing MO3.13 GALC KO1 cells and incubated for 48 h. Following this, transduced cells were selected by incubation for 48 h in medium containing 2 µg/mL puromycin. The pCW57 vector allows for addition of doxycycline to induce expression of GALC. Doxycycline titrations were used in combination with GALC activity assays (see below and *SI Appendix*, Fig. S3) to determine that 0.2 µg/mL doxycycline induced WT levels of GALC activity.

### Activity Assays.

GALC activity assays were modified from those described previously (81). MO3.13 cells were lysed in 10 mM Tris pH 8.0, 1 mM EDTA, 1% Triton X100, 140 mM NaCl, and 1 mM PMSF and precleared by incubation with protein A resin. Endogenous GALC protein was immunoprecipitated (IP) from cleared lysate using our monoclonal GALC antibodies described previously (82). Lysates were incubated at 4 °C overnight with GALC antibody and protein A resin. A control IP was carried out with protein A resin but with no GALC antibody. Beads were washed four times with lysis buffer before being transferred into activity assay buffer (20 mM sodium acetate, pH 4.6, 150 mM NaCl, 0.1% (v/v) NP40 containing fluorogenic substrate 0.3 mM 4-methylumbelliferyl β-D-galactopyranoside) preequilibrated to 37 °C. The reaction product was monitored at three timepoints over 6 h by fluorescence detection (excitation at 365 nm, emission at 445 nm) following addition of stopping buffer (480 mM NaOH, 380 mM glycine, pH 10.6).

### Lipidomics and Proteomics.

Details of lipidomics and proteomics methods including data collection and processing are provided in *SI Appendix*.

### Plasmids and Constructs for Protein Expression.

Amino acid numbering used throughout is based on the following sequences: human NFASC; UniProt ID: 094856, isoform 9 for NF155, and isoform 8 for NF186. Details of the amino acid composition of these isoforms and of the truncation constructs used in this study are provided in *SI Appendix*, Fig. S5 and Table S6.

### Protein Expression and Purification.

Codon-optimized NF155 and NF186 synthetic genes were purchased from GeneArt, and the relevant truncations cloned into the pHLSec expression vector using the *Age*I and *Kpn*I restriction sites. Constructs were sequence verified and then transiently transfected in HEK293-F cells, using the PEI MAX® (Polysciences, #24765) transfection reagent, as per the manufacturer’s instructions. The proteins were expressed for 4 d at 37 °C in a humidified 8% CO_2_ atmosphere. The media was harvested by centrifugation at room temperature, beginning with 100 g for 5 min to gently pellet and remove the cells, followed by centrifugation at 4,000 g for 10 min to further clarify the media before filtering through a 0.2-µm nylon membrane (Millipore). The media was then incubated with 1.5 mL Ni-NTA beads (Qiagen) at 4 °C on a rolling bed for 1 h. The Ni-NTA beads were captured using a gravity flow Econo-column (Biorad) and washed with wash buffer containing 50 mM HEPES pH 7.4, 150 mM NaCl, and 20 mM imidazole, and protein was eluted in elution buffer containing 50 mM HEPES pH 7.4, 150 mM NaCl, and 300 mM imidazole. The eluted protein was further purified via size exclusion chromatography in buffer containing 50 mM HEPES pH 7.4 and 150 mM NaCl. NF186 FL ECD, NF155 FL ECD, and NF155 IgG1-Fn2 were purified using a HiLoad 16/600 Superdex 200 pg column (Cytiva), and NF155 IgG1-4, NF186 IgG1-4, NF155 IgG5-Fn2, and NF155 Fn1-4 were purified using a HiLoad 16/600 Superdex 75 pg column (Cytiva).

To make deglycosylated FL NF155-ECD (NF155-DG), HEK293-F cells were transiently transfected in the presence of 5 µM kifunensine and purified as above, to yield the high mannose form (NF155-HM). This was then treated with 1 U Endo H_f_ (New England Biolabs) per 1 µg of NF155 and incubated at 30 °C for 3 h. The NF155-DG was separated from the Endo-H_f_ by loading onto a 5 mL HisTrap™ FF (Cytiva, #17528601) column in wash buffer and then eluting the NF155-DG in elution buffer. NF155-DG was then concentrated to 1.3 mg/mL and buffer exchanged into 50 mM HEPES pH 7.4 and 150 mM NaCl using an Amicon Ultra 30 kDa centrifugal concentrator (Millipore).

### Liposome-Binding Assays.

PC (#840051C), sulfatide (#131305P), 1,2-dimyristoyl-sn-glycero-3-phosphoethanolamine-*N*-(lissamine rhodamine B sulfonyl) (Rhod-PE) (#810157P), L-α-PS (#840032P), and D-galactosyl-α-1,1′ N-palmitoyl-D-erythro-sphingosine (GalCer) were purchased from Avanti. Ganglioside GM_4_ (GM4) was purchased from Merck (#345748). Lipids were dissolved/suspended in chloroform (aside from GalCer, which was dissolved in 2:1 chloroform:methanol), and the final desired mixtures were added to a glass vial before the solvent was evaporated under a nitrogen stream. To all liposomes prepared, 2% Rhod-PE was incorporated to aid with liposome visualization. The resultant lipid film was then hydrated using liposome buffer (50 mM HEPES pH 7.4 and 150 mM NaCl) to form multilamellar liposomes. A final liposome concentration of 0.8 mM in 50 µL was incubated with 1 µM protein, unless otherwise stated, for 30 min at room temperature with rotation. The liposomes were then pelleted by centrifugation at 20,000 g and the supernatant was removed. A further 50 µL aliquot of liposome buffer was added to the pellet and vortexed to wash the sample before a second centrifugation. The supernatant was removed once more and the pellet was resuspended in 10 µL of liposome buffer and 10 µL of 2× loading dye, separated using a NuPAGE 4 to 12% Bis-Tris gel (Invitrogen), stained with InstantBlue (Abcam), and imaged using a BioRad ChemiDoc MP imaging system. Densitometric analysis of gel images was performed using ImageJ and GelAnalyzer. For fitting of binding data, n = 4 independent experiments comparing binding of IgG1-Fn4 and IgG1-Fn2 were performed. For each replicate, the percentage of bound NF155 was fitted to the Hill equation using Prism version 9 (GraphPad), yielding values for B_max_, the Hill coefficient, and the percentage of sulfatide for half-maximal binding. Statistical tests to compare values for IgG1-Fn4 and IgG1-Fn2 were performed using a two-tailed paired *t* test in Prism, to control for liposome variability between experiments.

### Multi-Angle Light Scattering (MALS).

MALS experiments were performed immediately following SEC (SEC-MALS) by inline measurement of static light scattering (DAWN 8+; Wyatt Technology), differential refractive index (Optilab T-rEX; Wyatt Technology), and UV absorbance (1,260 UV; Agilent Technologies). 1 mg/mL samples of NF155 constructs (100 μL) were injected onto a Superdex 200 Increase 10/300 GL column (Cytiva) equilibrated in 50 mM HEPES pH 7.4, 150 mM NaCl at 0.4 mL/min. The molar masses of the major SEC elution peaks were calculated in ASTRA 6 (Wyatt Technology) using a protein dn/dc value of 0.185 mL/g. For determination of protein and glycan fractions, conjugate analysis was performed in ASTRA 6, using a glycan (modifier) dn/dc = 0.14 mL/g and theoretical UV extinction coefficient was calculated using ProtParam (83). Figures were prepared using Prism 9 (GraphPad).

### Negative Stain EM.

Purified NF155-ECD (10 nM) in 50 mM HEPES pH 7.4 and 150 mM NaCl was applied to glow-discharged 300 mesh formvar/carbon-coated copper grids (Agar Scientific) and stained with 2% (w/v) uranyl acetate using the side blotting method, as previously described (84). Electron micrograph images were collected using an FEI Tecnai G2 Spirit BioTWIN transmission electron microscope operating at 120 kV, equipped with an Ultrascan 1000X-U CCD camera (Gatan). Data collection was performed at 30,000 × nominal magnification (pixel size 3.31 Å) with a total electron dose of 20 to 40 e^−^/Å^2^ and −1.5 µm nominal defocus across 1 s exposures. In total, 95 images were taken and analyzed using CryoSPARC version 3.2.0. Images were CTF corrected, and 1,000 particles were picked manually, followed by template picking with an extraction box size of 150 pixels. From this, 21,703 particles were picked, generating 18 2D classes that were used for ab initio reconstruction of the NF155-ECD. This was followed by homogenous refinement with an initial low-pass resolution of 50 Å and a maximum align resolution of 10 Å. The overall resolution of the generated 3D map was calculated using the CryoSPARC Local Resolution tool with a Fourier Shell Correlation cutoff of 0.143 (85).

### Generating AF2 Models and Fitting to the EM Map.

Structural models were generated using a locally installed version of ColabFold version 1.3, implementing AF2 machine learning structure prediction, with default parameters (51, 52). AF2 models for the FL-ECD were positioned by rigid-body docking into the three-dimensional EM density map using the “fit in map” tool in UCSF ChimeraX (86). The C-terminal tail of the FL-ECD was removed from the model because it was predicted to be disordered (pLDDT < 50) and would thus not be resolved in the EM map. Correlation coefficients were calculated by generating maps around the docked atomic models, at the resolution of the EM map, and calculating map-to-map correlations in ChimeraX. Improved fitting of models to the EM map was carried out using molecular dynamics with flexible fitting (MDFF) as implemented by ISOLDE within ChimeraX (54). MDFF simulations used the AF2 model as a reference structure with strong distance and torsion restraints weighted using the PAE matrix and pLDDT values of the AF2 model, respectively. Following visual inspection of the model docked into the EM map, the IgG1-4 domain was manually repositioned by rotating to maximize the fit to density and MDFF simulations were repeated as above, maintaining the AF2 model as reference and retaining strong distance and torsion restraints.

### Fluorescent Liposome Clustering Assay.

Liposomes were prepared as detailed above. Additional lipids, 1,2-dioleoyl-sn-glycero-3-[(N-(5-amino-1-carboxypentyl)iminodiacetic acid)succinyl] (NiNTA-DGS) (#790404P) and 1,2-dimyristoyl-sn-glycero-3-phosphoethanolamine-N-(7-nitro-2-1,3-benzoxadiazol-4-yl) (NBD-PE) (#810143P), were purchased from Avanti® and dissolved in chloroform. Three lipid compositions (molar percentage) were made: 1) 4% NiNTA-DGS, 2% NBD, and 94% PC; 2) 50% sulfatide, 4% NiNTA-DGS, 2% NBD-PE, and 44% PC; and 3) 50% sulfatide, 2% Rhod-PE, 48%. Samples containing 0.4 mM liposomes with compositions 1 or 2 (as detailed above) were preincubated with 20 nM NF155-ECD in liposome buffer at room temperature with rotation for 15 min. Liposome composition 3 was added (0.4 mM) to each and incubated for a further 15 min. Samples were diluted 10-fold and placed on a microscope slide, under a coverslip. Liposomes were imaged using the EVOS M5000 Imaging System at 20× magnification. Colocalization of the pink and green liposomes was quantified using the ImageJ coloc2 software.

## Supplementary Material

Appendix 01 (PDF)

Dataset S01 (XLSX)

## Data Availability

The mass spectrometry proteomics data produced in this study have been deposited to the ProteomeXchange Consortium via the PRIDE repository (87) (https://www.ebi.ac.uk/pride/) with the dataset identifiers PXD036727 and 10.6019/PXD036727 (for the MO3.13 cell lines), and PXD039341 and 10.6019/PXD039341 (for the i3N cell lines). Lipidomics data are supplied with this submission as a supplementary file. AF2 models and associated statistics have been deposited in the University of Cambridge Data Repository (10.17863/CAM.93310). Raw EM images are deposited in the EM Public Image Archive EMPIAR with accession code EMPIAR-11400 (88), and processed data are deposited in the EM Data Bank with accession code EMD-16540 (89). The final MDFF-refined AF2 model of NF155-ECD is deposited in the University of Cambridge Data Repository (10.17863/CAM.93310).
