# Supplementary material for: Altered plasma membrane abundance of the sulfatide-binding protein NF155 links glycosphingolipid imbalances to demyelination
Source: Proc Natl Acad Sci U S A. 2023 Mar 30;120(14):e2218823120. doi: 10.1073/pnas.2218823120 (PMC10083573; doi:10.1073/pnas.2218823120)
Supplement: Supplementary file 1 — Appendix 01 (PDF) [file pnas.2218823120.sapp.pdf]

Supporting Information for:

Altered plasma membrane abundance of the sulfatide-binding protein NF155 links glycosphingolipid imbalances to demyelination.

Authors: Shannon J. McKie, Alex S. Nicholson, Emily Smith, Stuart Fawke, Eve Caroe, James C. Williamson, Benjamin G. Butt, Denisa Kolářová, Ondřej Peterka, Michal Holčapek, Paul J. Lehner, Stephen C. Graham and Janet E. Deane

Corresponding author: Janet Deane

Email: jed55@cam.ac.uk

This PDF file includes:

Figures S1 to S16

Tables S1 to S6

Supporting Methods

SI References

Other supporting materials for this manuscript include the following:

Lipidomics Dataset

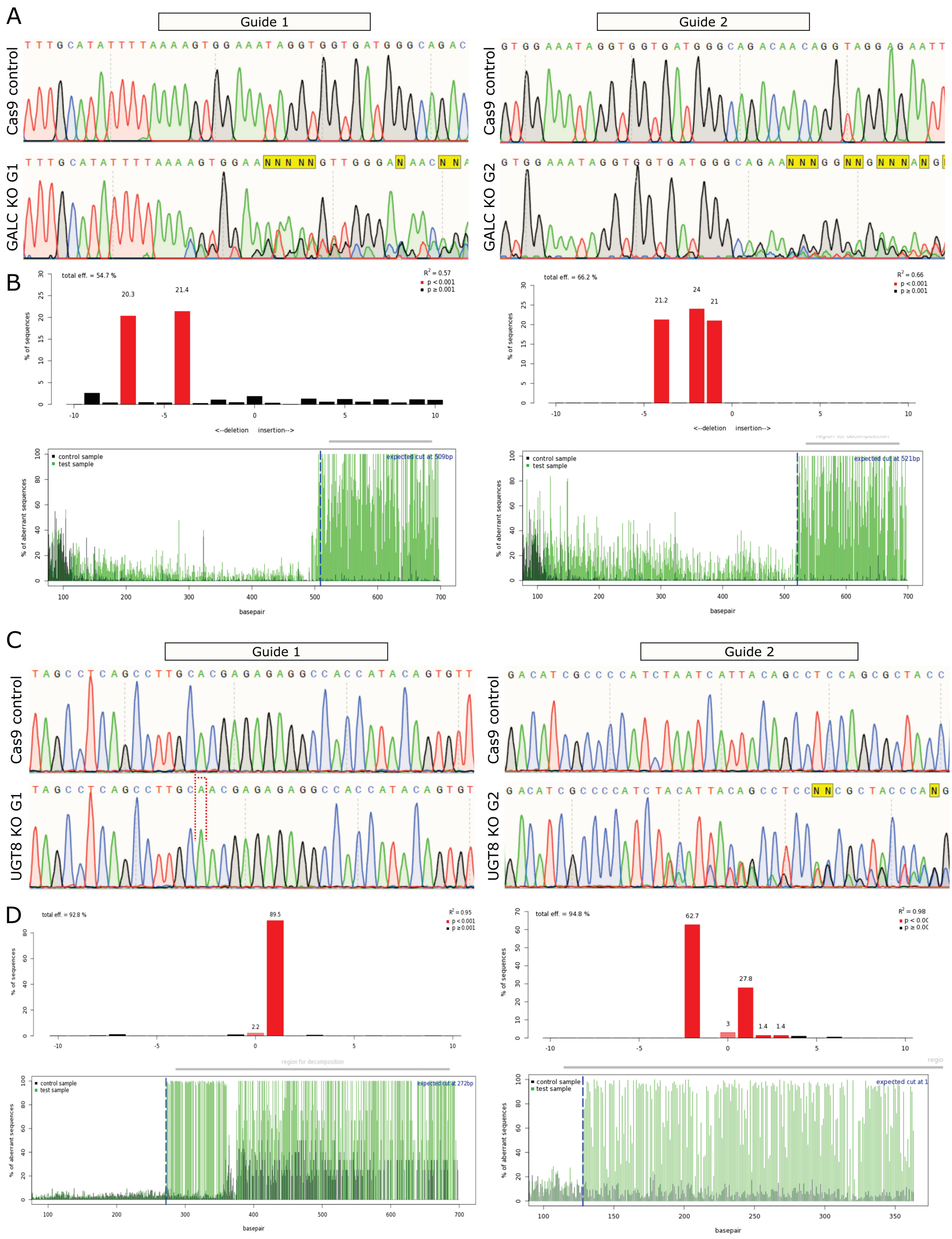

Figure S1. Confirmation of editing at gRNA-targeted sites of GALC and UGT8 genes. (A) DNA sequencing chromatograms are shown across the relevant region of the genome for cell lines transfected with untargeted Cas9 (Cas9 control, *top*), showing the unedited sequence, and for Cas9 targeted by sgRNA1 and sgRNA2 to the GALC gene (*bottom*) in the clonal MO3.13 lines used in this study. Overlapping, altered sequences demonstrate editing at the target site. **(B)** Analysis of sequence chromatograms using TIDE (1), predicting the frequency and position of deletions and insertions as a result of CRISPR/Cas9 editing. **(C and D)** As for panels (A and B) but for the UGT8 KO clonal cell lines.

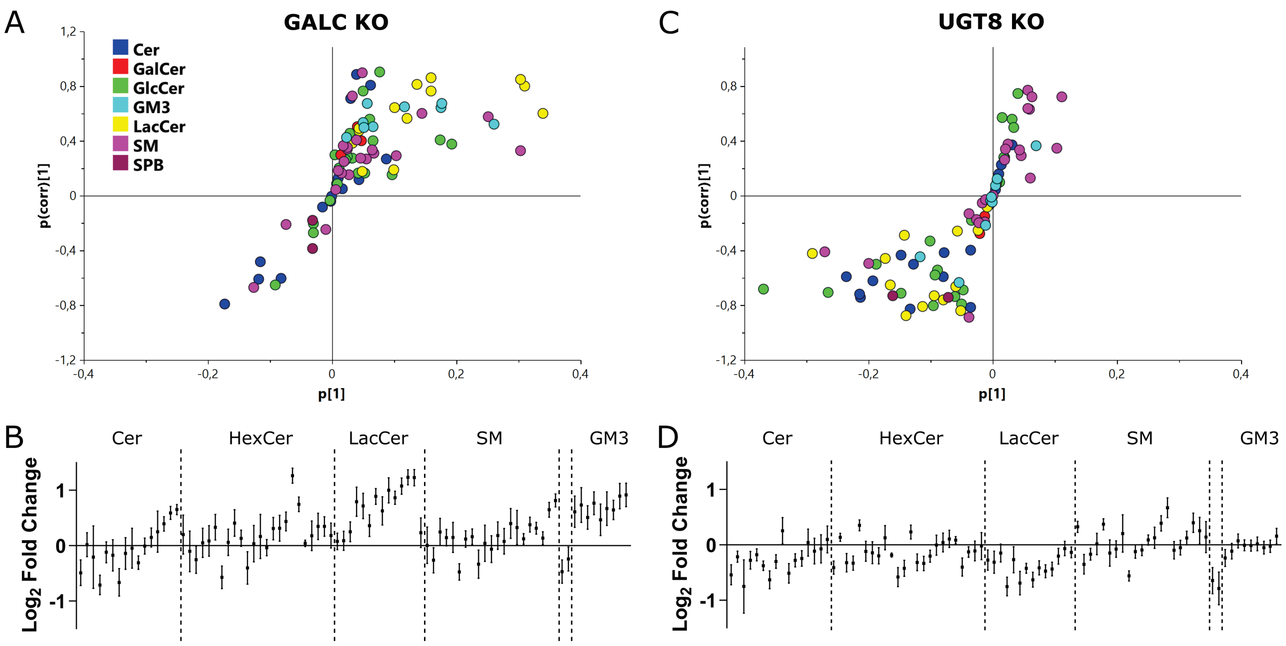

Figure S2. Targeted lipidomics quantifying ceramide, sphingomyelin and glycosylated sphingolipids. (A) S-plot demonstrating the up-regulated (upper right corner) and down-regulated (lower left corner) sphingolipid species comparing GALC KO samples (n=3 for each of two cell lines) with Cas9 control cells (n=3). **(B)** The same lipidomics data as in (A) analysed by lipid class. The log base-2 fold change in abundance between GALC KO cells and Cas9 control cells of all quantified sphingolipids. Samples (n=3) for each of the two GALC KOs were included in the analysis against Cas9 cells (n=3). Mean and standard error are plotted for each lipid species. **(C)** S-plot analysis as in (A) for UGT8 KO cell lines. **(D)** Quantification by lipid species as in (B) for UGT8 KO cell lines.

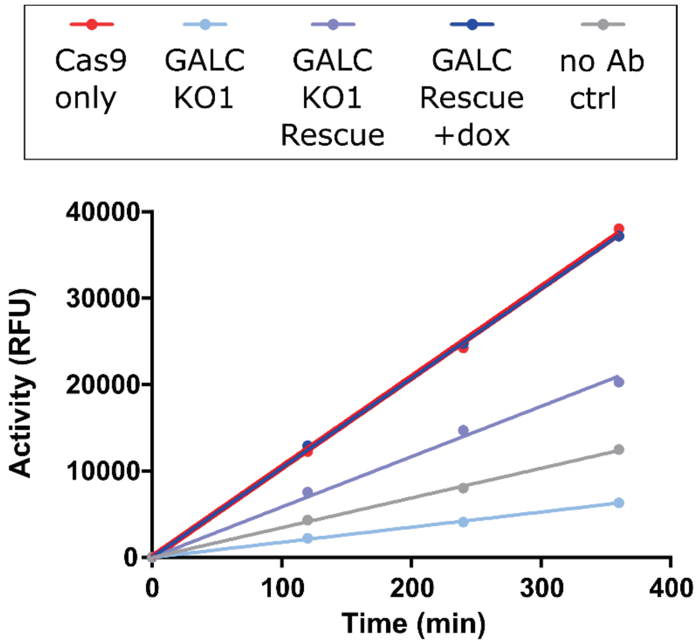

Figure S3. Activity assays for the control, GALC KO and GALC rescue cell lines. GALC activity assays following immunoprecipitation (IP) from control, GALC KO1 and GALC KO1 Rescue cell lines in the absence and presence of doxycycline (GALC expression in the rescue cell line is controlled by a doxycycline-inducible promoter). Low levels of GALC activity are present in the rescue cell line in the absence of dox, but with the addition of 0.2 µg/mL dox GALC expression is restored to control (Cas9 only) activity levels. A no antibody (no Ab) control IP is also included.

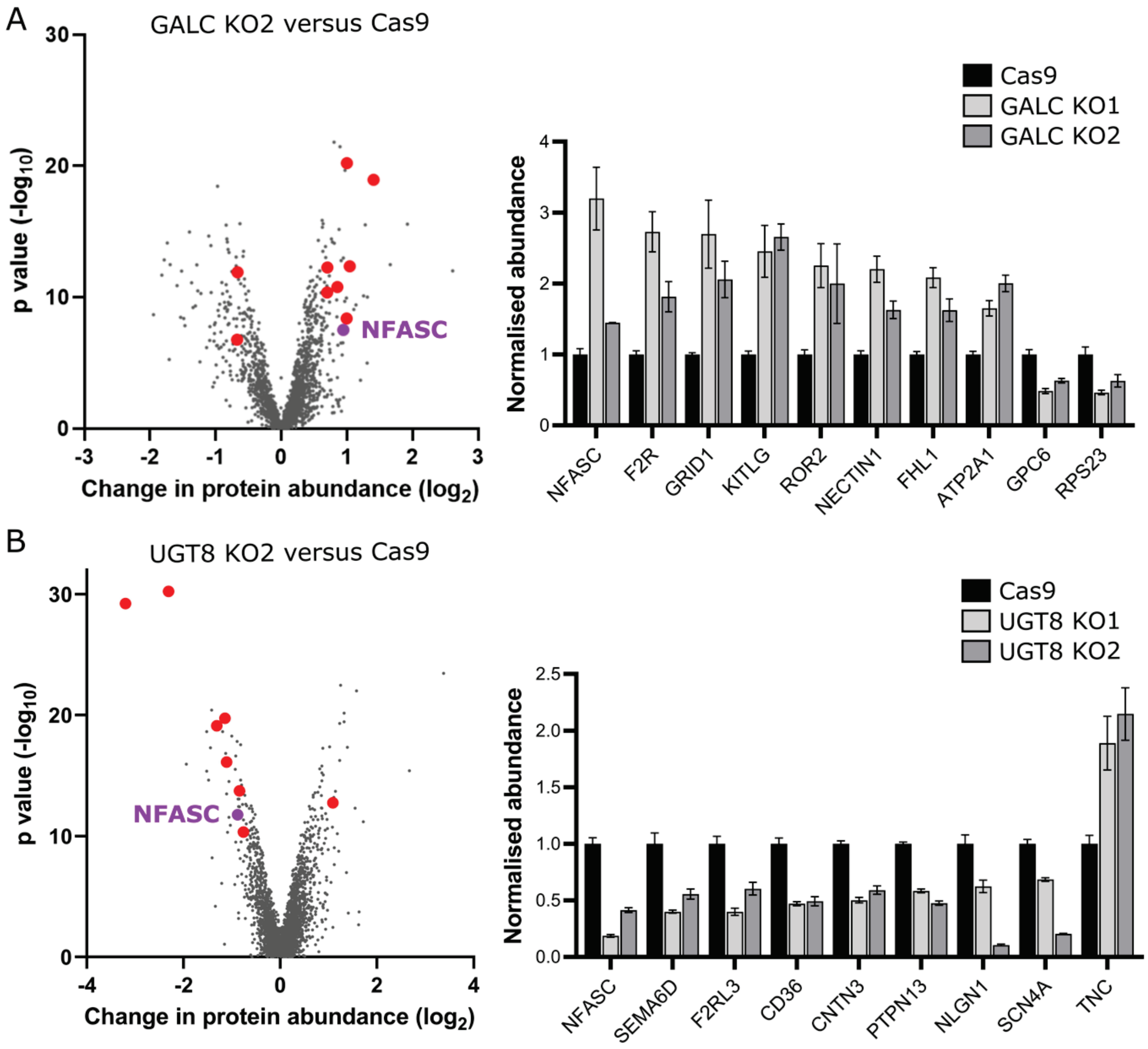

Figure S4. PMP-MS data for additional clonal GALC and UGT8 KO cell lines. (A) Quantitative mass spectrometry following enrichment of plasma membrane proteins (PMP-MS) from the GALC KO2 cell line compared with the Cas9 control. *Left*: volcano plot with the horizontal axis showing average fold change across three biological replicates and the vertical axis showing significance (two-sided t test) across the three replicates. The ten high-confidence targets (criteria as detailed in the main text) are coloured in red with NFASC specifically highlighted in purple. *Right*: Normalised protein abundance values, from the PMP-MS data, for high-confidence targets in both the GALC clonal KO cell lines. Data shown in Table S1. Normalised abundances have been used to allow comparison across genes with different absolute abundances in the cell. **(B)** As for panel (A) but for the UGT8 KO2 cell line. Data shown in Table S2.

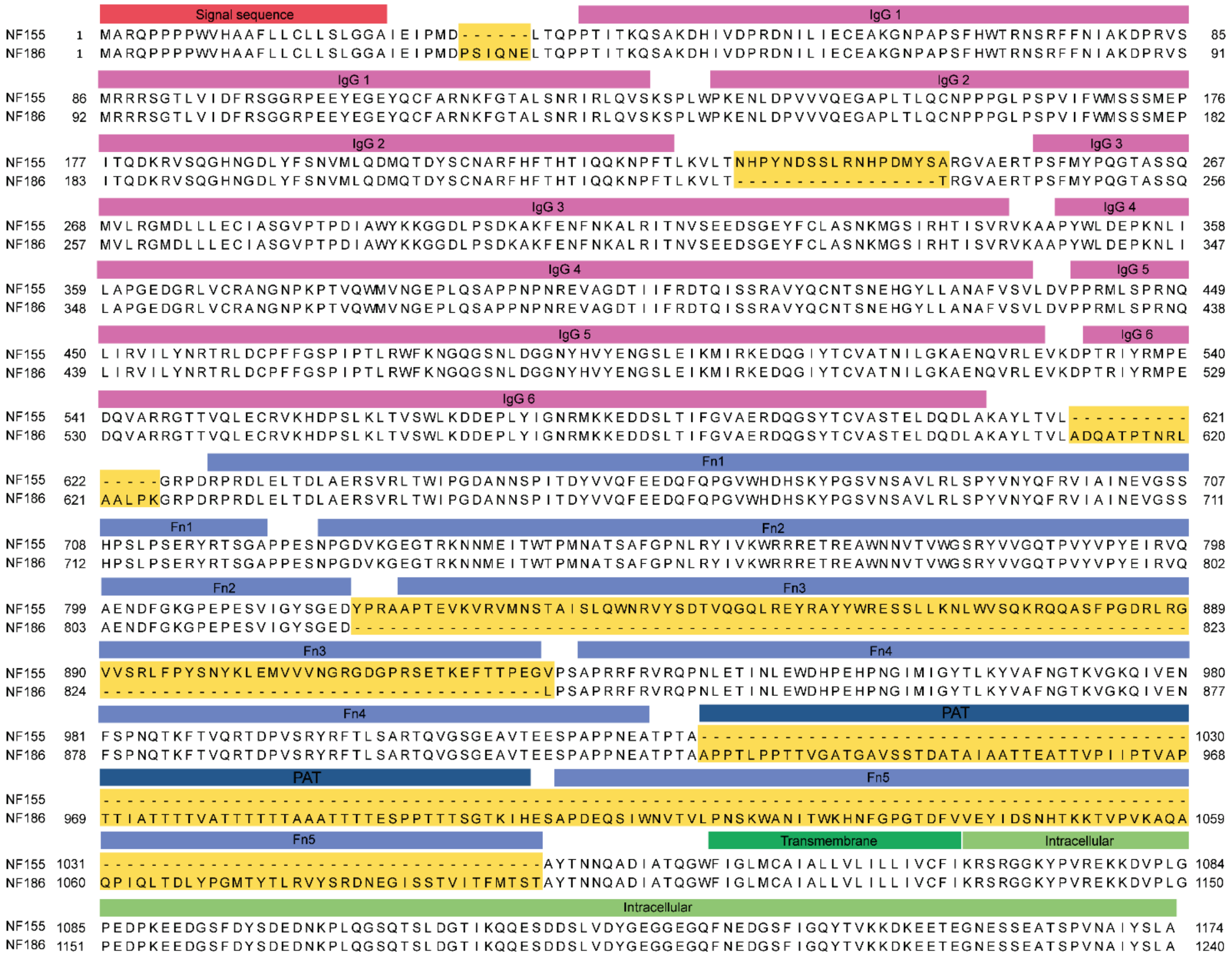

Fig. S5. Sequence composition of NF155 and NF186 isoforms. IgG-like domains highlighted in pink, Fn-like domains in light blue, PAT domain in dark blue, transmembrane domain in dark green, intracellular domain in light green and differences between the two isoforms in yellow. Alignment generated using Jalview (2) and annotated in Adobe Illustrator.

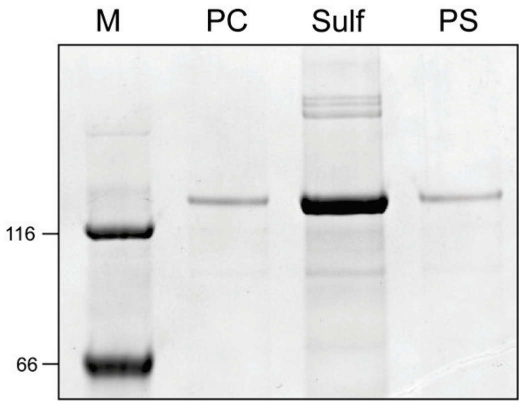

Fig. S6. NF155 does not bind the anionic lipid phosphatidylserine (PS). Liposome pull down assay demonstrating that NF155-ECD does not non-specifically bind negatively charged membrane, but specifically binds sulfatide. Here, three liposome compositions were tested for binding to 1 µM NF155 FL ECD: 98% PC: 2% Rhod-PE (lane PC), 28% PC: 2% Rhod-PE: 70% sulfatide (Sulf), and 28% PC: 2% Rhod-PE: 70% PS (lane PS). Samples separated by SDS PAGE and stained using Coomassie.

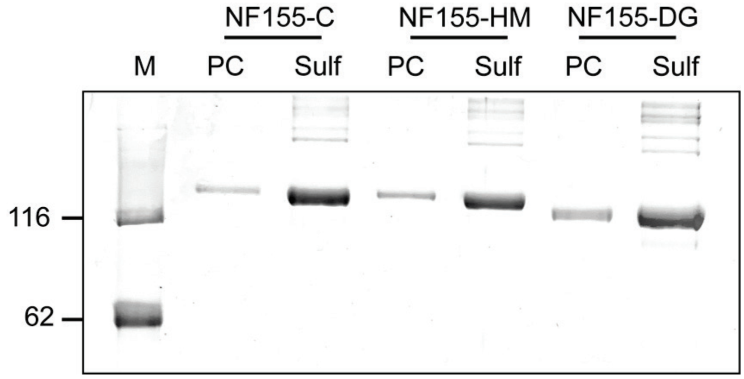

Fig S7. Deglycosylated NF155 retains binding to sulfatide. Liposome pull down assay demonstrating that the glycosylation of NF155 FL ECD does not affect sulfatide binding. Liposomes used to test binding of 1 µM NF155 FL ECD were 98% PC: 2% Rhod-PE (PC), or 48% PC: 2% Rhod-PE: 50% sulfatide (Sulf). NF155-C has complex glycosylation, NF155-HM has high mannose glycosylation and NF155-DG is deglycosylated. Samples separated by SDS PAGE and stained using Coomassie. Due to the low stability of NF155 when deglycosylated this experiment was performed immediately following EndoH treatment.

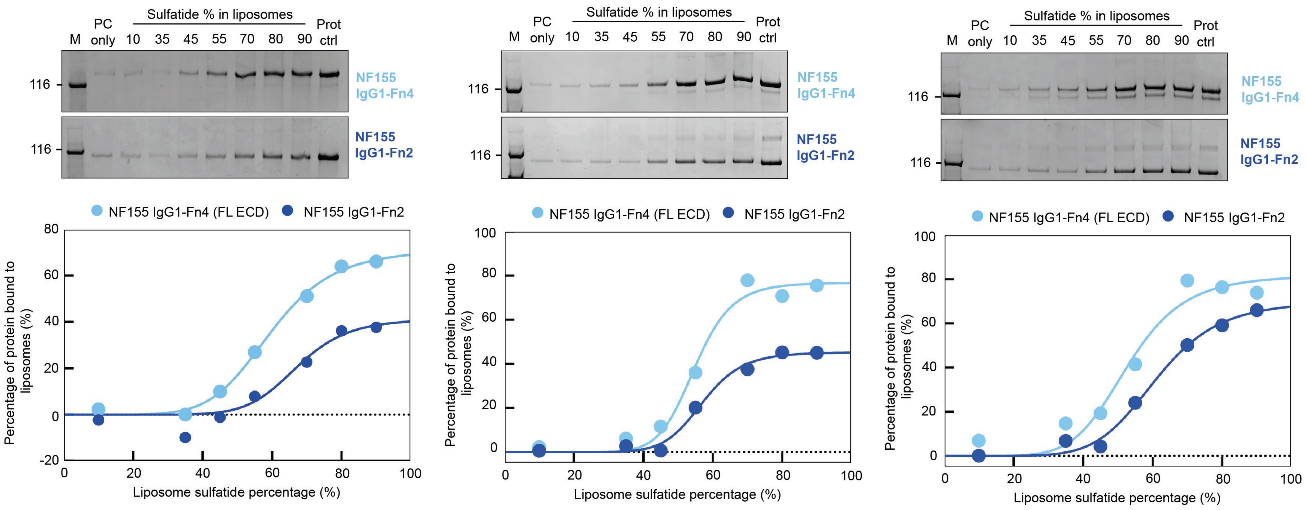

Figure S8. Replicates of quantitive liposome binding data for NF155 IgG1-Fn4 and IgG1-Fn2. Three independent replicates of the data shown in Fig. 2D of the main text are displayed as follows. *Top:* Liposome binding assay performed with 250 nM NF155 using 1.6 mM liposomes containing increasing concentrations of sulfatide (IgG1-Fn4, top gel, IgG1-Fn2, lower gel). *Bottom:* Densitometric analysis of the SDS PAGE reveals a sigmoidal binding relationship for both NF155 constructs (IgG1-Fn4, light blue, IgG1-Fn2, dark blue). Prot ctrl represents total protein added to each assay sample. A total of n=4 independent experiments were performed.

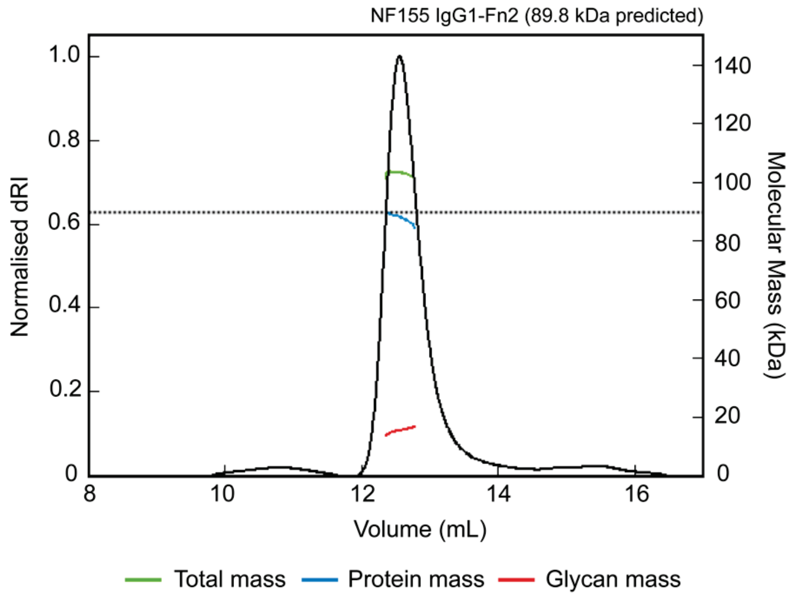

Fig. S9. Size exclusion chromatography coupled to multi-angle light scattering (SEC-MALS) for the NF155 truncation IgG1-Fn2. Protein conjugate mass analysis of the elution peak demonstrates that NF155 IgG1-Fn2 is monomeric with a calculated protein mass of 87.6 kDa (consistent with the predicted mass of 89.8 kDa, dotted line) and 15.3 kDa glycans.

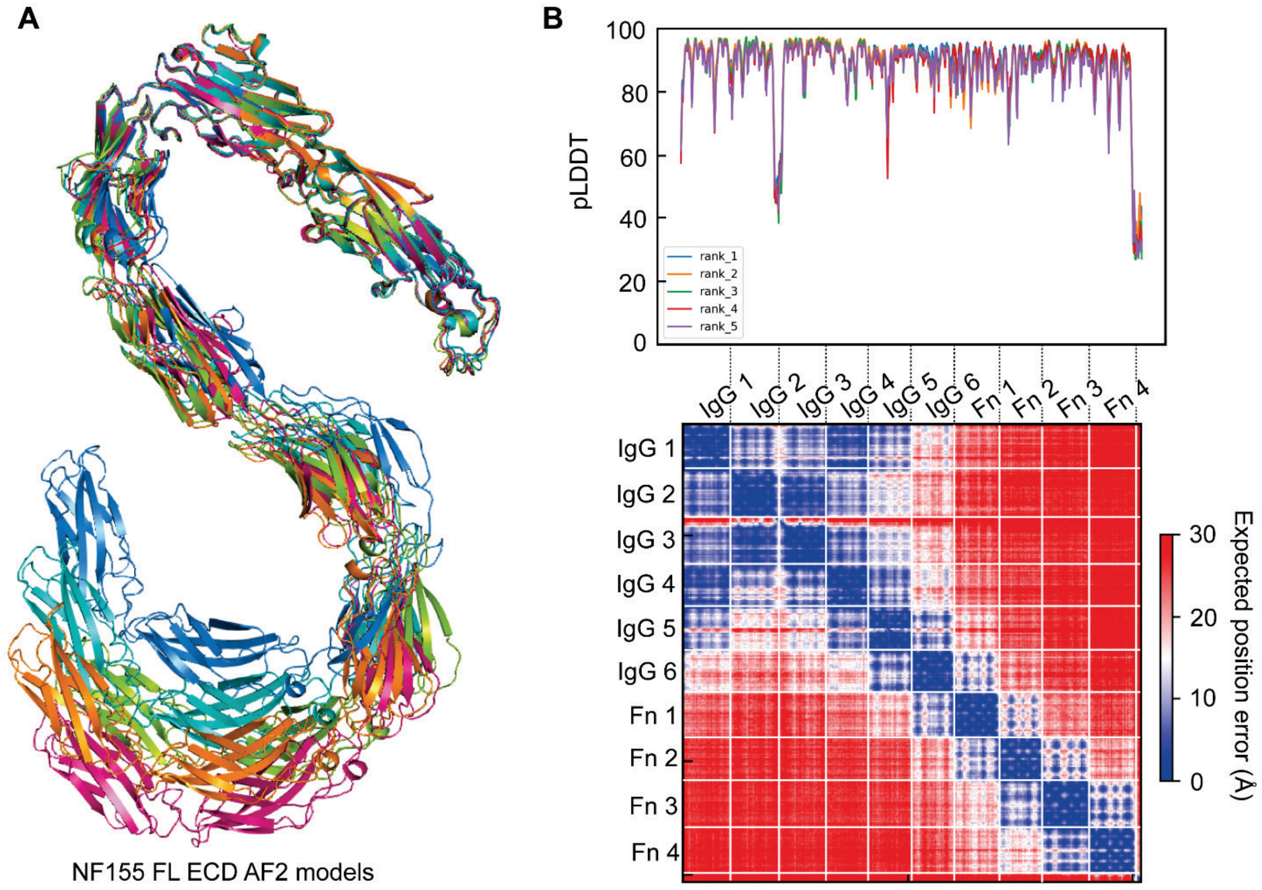

Fig. S10. AF2 models of the NF155-ECD. (A) Ribbon diagrams of the five AF2 predictions of the NF155 FL ECD aligned using the four N-terminal domains, IgG1-IgG4. **(B)** *Top,* pLDDT plots demonstrating good per-residue confidence for all five NF155 FL ECD predictions. *Bottom*, Representative Predicted Aligned Error (PAE) plot for NF155 FL ECD, with domain boundaries marked (white lines), demonstrating the low confidence (high error, red) of long-distance predictions, aside from the four N-terminal domains, IgG1-IgG4.

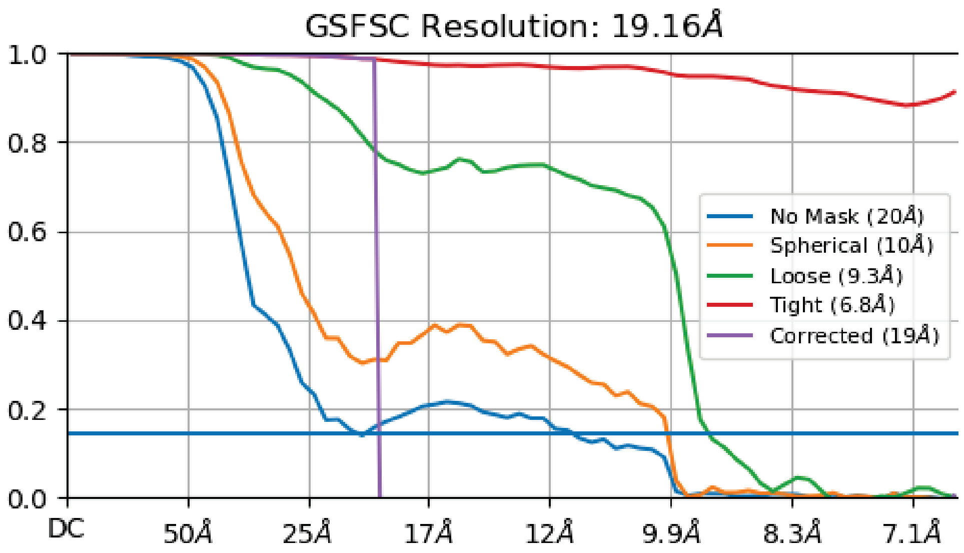

Fig. S11. Resolution estimation of the 3D reconstruction of NF155-ECD based on the Fourier Shell Correlation (FSC)=0.143 criterion with different masking options (3). Performed using the CryoSPARC Local Resolution tool.

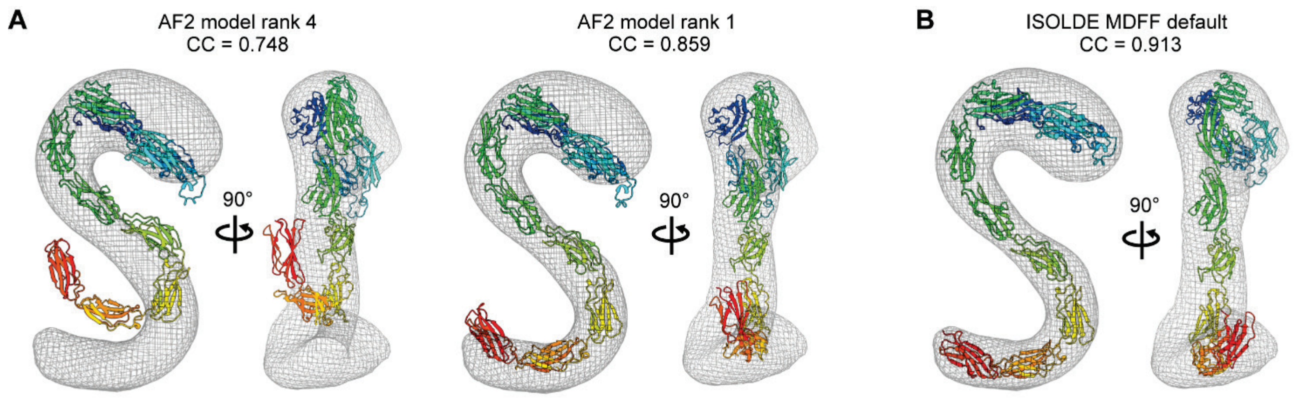

Fig. S12. Initial docking of AF2 models of the NF155-ECD into negative-stain EM maps. (A) Rigid-body fits into the negative stain EM map of AF2 models with the lowest (*left*) and highest (*right*) correlation coefficient (CC) when compared to the experimental map. Fit and CC calculations performed using Chimera-X (4). **(B)** The AF2 model rank 1 following molecular dynamics flexible fitting (MDFF) implemented using ISOLDE (5) with strong torsion and distance restrains weighted by the AF2 model pLDDT and PAE scores, respectively. For each representative image two orientations are shown rotated by 90° about the y-axis demonstrating the overall fit to the map.

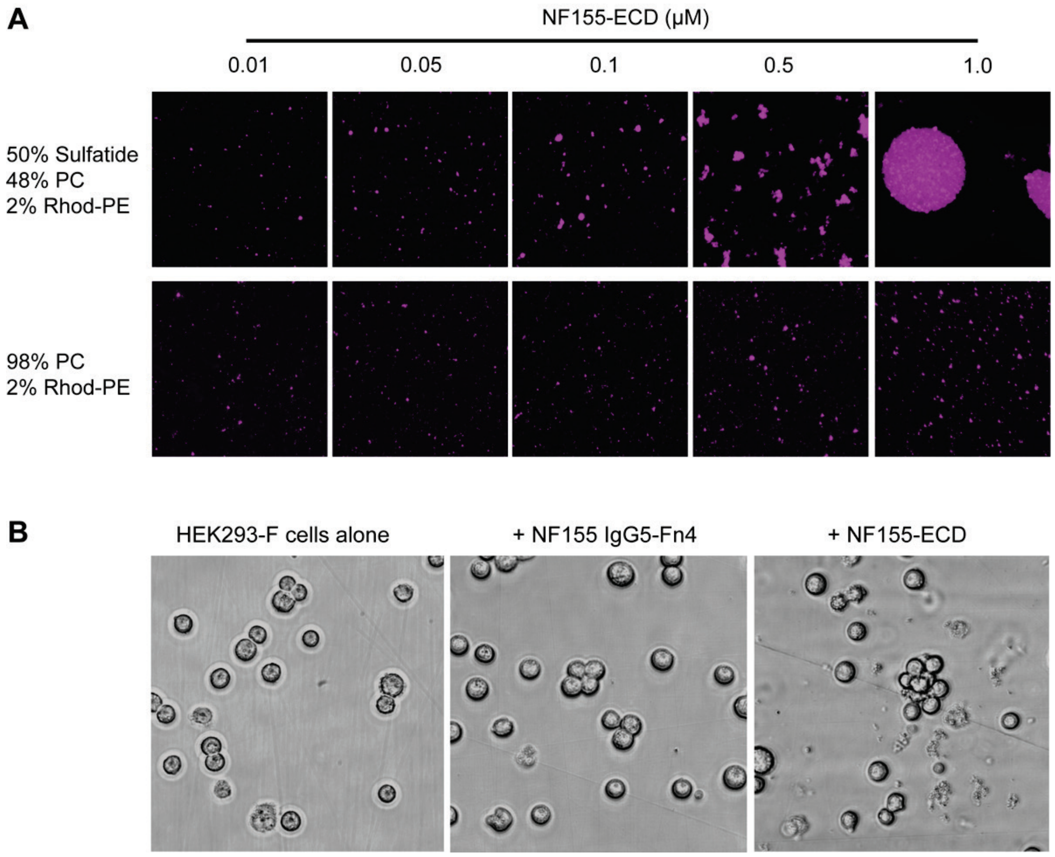

Fig. S13. NF155-ECD causes liposome aggregation and cellular membrane disruption. (A) Incubation of increasing concentrations of NF155-ECD with sulfatide-containing liposomes, caused increasingly severe liposome aggregation, while incubation with liposomes without sulfatide had no effect. **(B)** Incubation of NF155-ECD with HEK293-F cells caused increased cell clustering, membrane blebbing and loss of intracellular material, while incubation with the NF155 N-terminal truncation (IgG5-Fn4) did not.

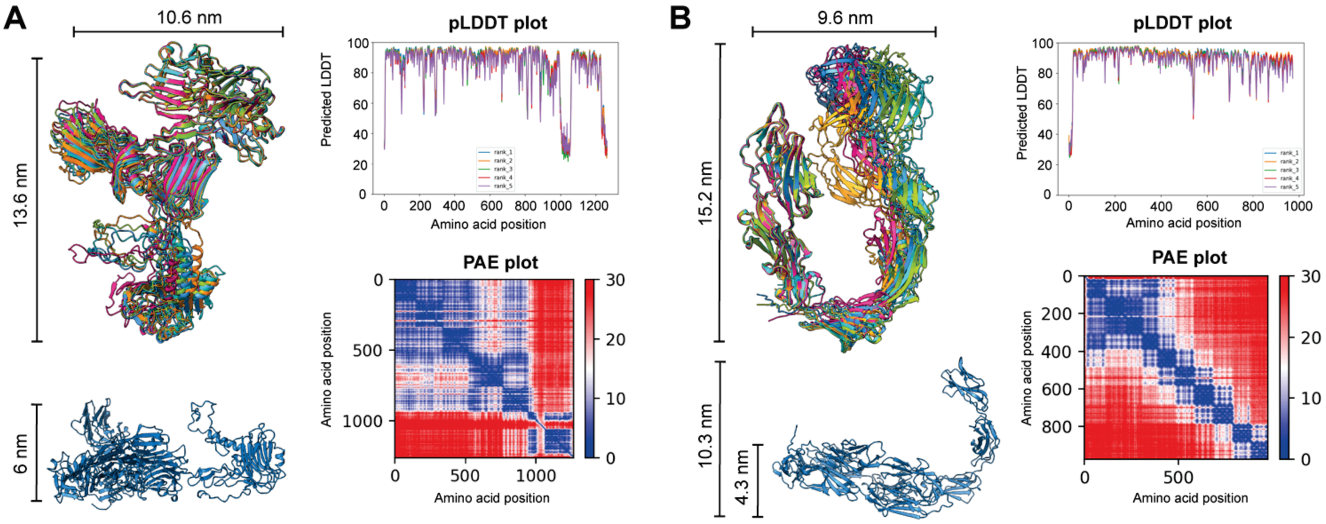

Fig. S14. AF2 models of CNTN1 and Caspr. (A) Ribbon diagram of the five Caspr ECD predictions aligned using amino acids 1-528, with dimensions, and the corresponding pLDDT and a representative PAE plot from predictions rank 1 (blue) (B) Ribbon diagram of the five CNTN1 ECD predictions aligned using amino acids IgG domains 1-4, with dimensions, alongside the corresponding pLDDT plot and a representative PAE plot from prediction rank 1 (blue).

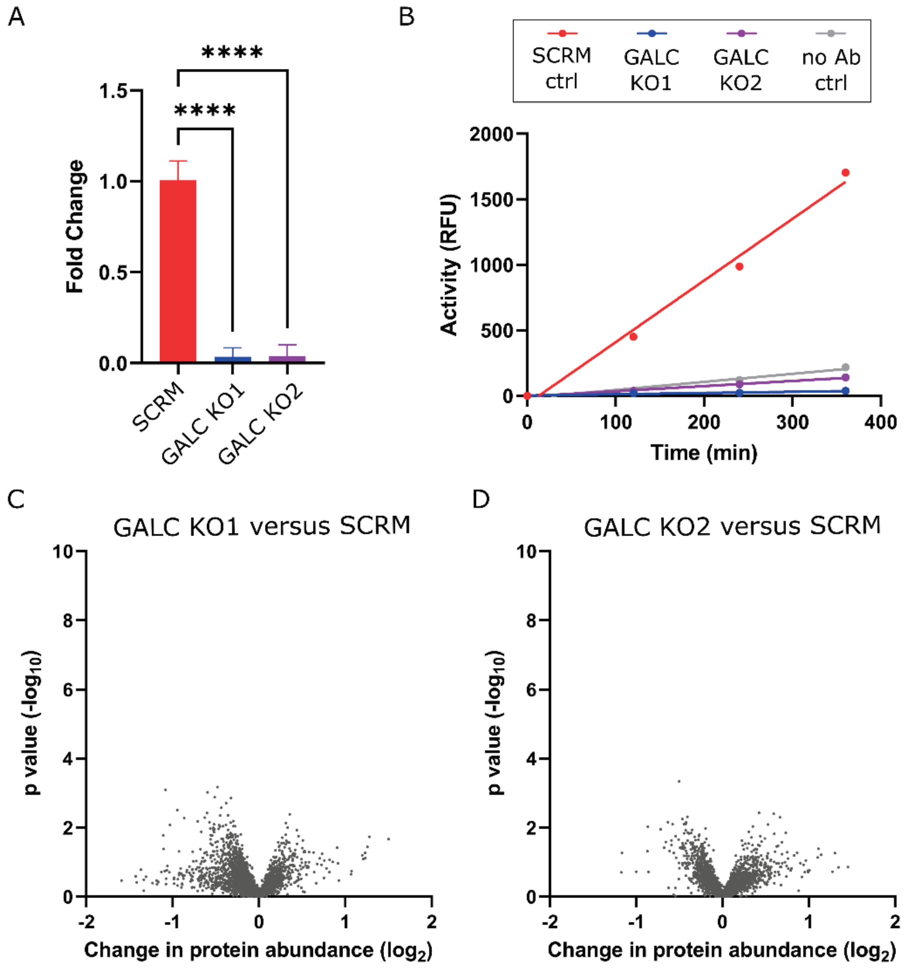

Fig. S15. Characterisation of GALC KO in i3N neuronal cells. (A) Quantitiative PCR of GALC gene expression in i3N neuronal cell lines expressing guide RNAs targeting dCas9 to the GALC gene (GALC KO1 and KO2) or control scrambled (SCRM) guide RNA. Data include technical and biological triplicates for all samples. **** $p < 0.0001$. **(B)** GALC activity assay data from i3N neuronal cell lines. Data presented are as described in Fig. S3. **(C)** and **(D)** PMP-MS data comparing GALC KO1 and KO2 to SCRM control cell lines. For both GALC KO1 and KO2 there were zero target genes identified with *q*-values < 0.05. Note the difference in scale of the axes compared with Figures 1C and 1E.

A Mass spectrometry method

Start Time (min): **0**
End Time (min): **190**
Cycle Time (sec): **3**

Master Scan:
- MS OT
  - Detector Type: **Orbitrap**
  - Orbitrap Resolution: **120000**
  - Mass Range: **Normal**
  - Use Quadrupole Isolation: **True**
  - Scan Range (m/z): **400-1500**
  - RF Lens (%): **60**
  - AGC Target: **Custom**
  - Normalized AGC Target (%): **125**
  - Maximum Injection Time Mode: **Custom**
  - Maximum Injection Time (ms): **50**
  - Microscans: **1**
  - Data Type: **Profile**
  - Polarity: **Positive**
  - Source Fragmentation: **Disabled**
  - Scan Description:

Filters:
- MIPS
  - Monoisotopic Peak Determination: **Peptide**
- Charge State
  - Include charge state(s): **2-7**
  - Include undetermined charge states: **False**
- Dynamic Exclusion
  - Use Common Settings: **False**
  - Exclude after n times: **1**
  - Exclusion duration (s): **90**
  - Mass Tolerance: **ppm**
  - Low: **10**
  - High: **10**
  - Exclude Isotopes: **True**
  - Perform dependent scan on single charge state per precursor only: **True**
- Intensity
  - Filter Type: **Intensity Threshold**
  - Intensity Threshold: **5.0e3**

- Data Dependent
  - Data Dependent Mode: **Cycle Time**
  - Time between Master Scans (sec): **3**

- Scan Event Type 1:
  - Scan:
  - $ddMS^2$ IT CID
    - Isolation Mode: **Quadrupole**
    - Isolation Window (m/z): **0.7**
    - Isolation Offset: **Off**
    - Activation Type: **CID**
    - Collision Energy Mode: **Fixed**
    - CID Collision Energy (%): **35**
    - CID Activation Time (ms): **10**
    - Activation Q: **0.25**
    - Multistage Activation: **False**
    - Detector Type: **Ion Trap**
    - Ion Trap Scan Rate: **Rapid**
    - Mass Range: **Normal**
    - Scan Range Mode: **Auto**
    - AGC Target: **Custom**
    - Normalized AGC Target (%): **80**
    - Maximum Injection Time Mode: **Auto**
    - Microscans: **1**
    - Data Type: **Centroid**
    - Scan Description:

  Filters:
  - Precursor Selection Range
    - Selection Range Mode: **Mass Range**
    - Mass Range (m/z): **400-2000**
  - Precursor Ion Exclusion
    - Exclusion mass width: **m/z**
    - Low: **19**
    - High: **7**
  - Isobaric Tag Loss Exclusion
    - Reagent: **TMT**

  - Data Dependent
    - Data Dependent Mode: **Scans Per Outcome**

  - Scan Event Type 1:
    - Scan:
    - $ddMS^3$ OT HCD
      - MS^n Level: **3**
      - Synchronous Precursor Selection: **True**
      - Number of SPS Precursors: **10**
      - MS Isolation Window (m/z): **2**
      - MS2 Isolation Window (m/z): **2**
      - Isolation Offset: **Off**
      - Activation Type: **HCD**
      - HCD Collision Energy (%): **65**
      - Detector Type: **Orbitrap**
      - Orbitrap Resolution: **50000**
      - Mass Range: **Normal**
      - Scan Range Mode: **Define m/z range**
      - Scan Range (m/z): **100-1000**
      - AGC Target: **Custom**
      - Normalized AGC Target (%): **40**
      - Maximum Injection Time Mode: **Custom**
      - Maximum Injection Time (ms): **120**
      - Microscans: **1**
      - Data Type: **Profile**
      - Use EASY-IC™: **False**
      - Scan Description:
      - Number of Dependent Scans: **3**

B Mass spectrometry searching method

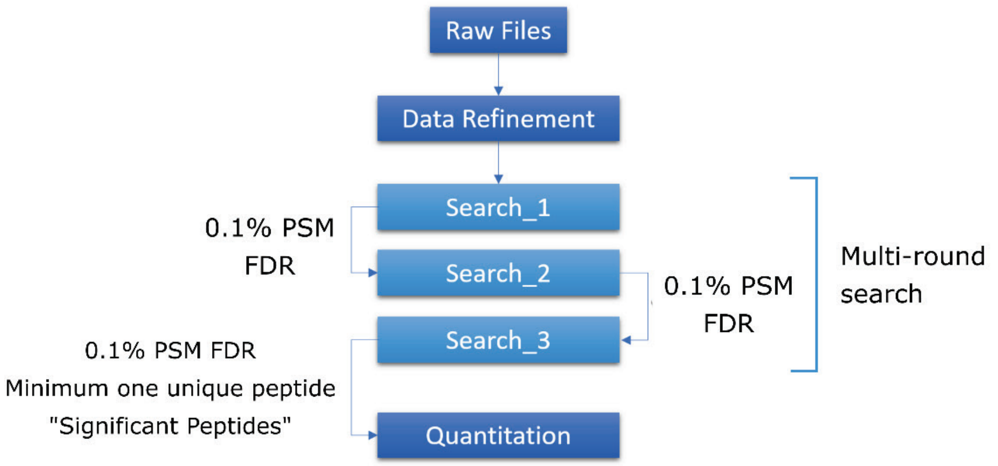

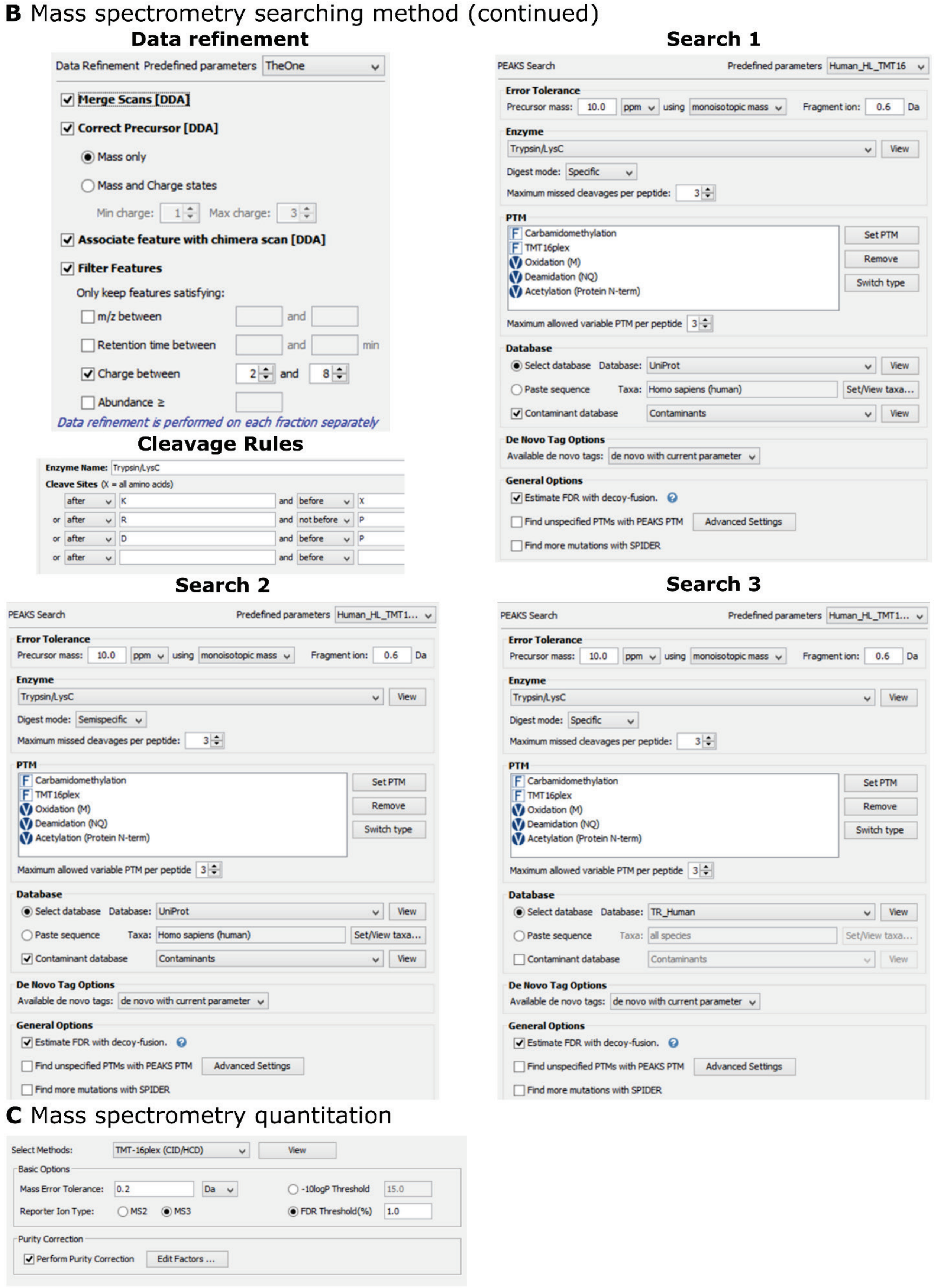

Fig. S16. Collection and processing of protein mass spectrometry data. Parameters for protein mass spectrometry data collection **(A)**, data searching **(B)** and quantitation **(C)** for data shown in **Figure 1**.

Table S1. High confidence targets identified in PMP-MS of GALC cell lines compared with Cas9 control cells.

Gene ID	Description	#Unique peptides	GALC KO1/Cas9		GALC KO2/Cas9		GALC Rescue/GALC KO1	
			Log2 Fold change	Significance q-value	Log2 Fold change	Significance q-value	Log2 Fold change	Significance q-value
NFASC	Neurofascin	25	1.677	1.12E-05	0.535	2.40E-02	-0.608	2.39E-02
F2R	Proteinase-activated receptor 1	3	1.449	1.13E-04	0.858	6.80E-03	-1.027	2.85E-03
GRID1	Glutamate receptor delta-1	12	1.430	1.34E-04	1.046	3.51E-03	-1.256	9.60E-04
KITLG	Kit ligand	5	1.295	1.56E-04	1.410	2.36E-04	-1.273	7.62E-04
ROR2	Tyr-kinase TM receptor	6	1.171	4.83E-03	0.999	2.02E-02	-0.907	3.61E-02
NECTIN1	Nectin-1	17	1.139	4.50E-05	0.705	3.63E-03	-0.938	7.61E-04
FHL1	Four and a half LIM domain 1	2	1.059	3.91E-04	0.699	8.39E-03	-1.073	9.88E-04
ATP2A1	Sarcoplasmic/ER Ca ATPase 1	19	0.723	5.60E-04	1.001	1.36E-04	-0.474	1.31E-02
GPC6	Glypican-6	4	-1.045	8.75E-05	-0.665	4.18E-03	0.503	2.41E-02
RPS23	40S ribosomal protein S23	3	-1.110	2.11E-03	-0.672	4.61E-02	0.937	1.00E-02

Table S2. High confidence targets identified in PMP-MS of UGT8 KO cell lines compared with Cas9 control cells.

Gene ID	Description	#Unique peptides	UGT8 KO2/Cas9		UGT8 KO3/Cas9	
			Log2 Fold change	Significance q-value	Log2 Fold change	Significance q-value
SEMA6D	Semaphorin-6D	3	-1.310	4.11E-04	-2.530	1.72E-05
NFASC	Neurofascin	18	-0.880	1.44E-02	-1.390	1.72E-03
F2RL3	Proteinase-activated receptor 4	3	-0.760	2.57E-02	-1.350	1.70E-03
CD36	Platelet glycoprotein 4	13	-1.110	1.79E-03	-1.080	1.74E-03
CNTN3	Contactin-3	5	-0.840	6.26E-03	-1.060	1.72E-03
PTPN13	Tyr phosphatase non-receptor 13	12	-1.140	3.54E-04	-0.770	2.71E-03
NLGN1	Neuroligin-1	4	-3.200	3.37E-06	-0.620	4.42E-02
SCN4A	Sodium channel type 4 subunit a	13	-2.310	3.36E-06	-0.590	1.63E-02
TNC	Tenascin	4	1.090	9.72E-03	0.840	2.82E-02

Table S3. sgRNA target sequences

Gene	Exon	Target sequence
**GALC**	3	5' - AAAGTGGAAATAGGTGGTGA
**GALC**	3	5' - GGTGGTGATGGGCAGACAAC
**UGT8**	2	5' - ATGGTGGCCTCTCTCGTGCA
**UGT8**	2	5' - TGGAGGCTGTAATGATTAGA

Table S4. Sequencing primers across genomic edit sites

Gene	Fwd Primer 5' -> 3'	Rev Primer 5' -> 3'
**GALC**	GCCCTACTTGCCCAATGATTGGCACAGAAAG	CAAAACTGTCCCTATGCTCTGTCCTGTATATATAG
**UGT8**	TGCTGTTGGGATAGCGAAGG	CACAAATCCACACATATCATTAGGG

Table S5. Composition of internal standard mixture for lipidomics analysis. Stock solutions of individual internal standards were prepared in the mixture of CHCl3/MeOH (1:1, v/v).

Lipid class	Internal standard	Molecular Weight	Stock solution [mg/mL]	V [µL]	Concentration [pmol/mg of cells]
SM	SM 18:1;O2/18:1-D9	737.6391	1	96	17.35
	SM 18:1;O2/12:0	646.5049	2	42	17.32
Cer	Cer 18:1-D7;O2/18:0	572.5868	1	12	2.79
	Cer 18:1;O2/17:0	551.5277	2	6	2.90
	Cer 18:1;O2/12:0	481.7900	2	5.1	2.82
GlcCer	GlcCer 18:1-D5;O2/18:0	732.6270	1	18	3.28
	GlcCer 18:1;O2/12:0	643.9350	0.2	79.5	3.29
GalCer	GalCer 18:1-D7;O2/13:0	664.5600	0.004	420	0.34
	GalCer 18:1;O2/12:0	643.5020	0.004	405	0.34
LacCer	LacCer 18:1-D7;O2/15:0	854.6455	1	6	0.94
	LacCer 18:1;O2/12:0	805.5551	2	3	0.99
Ga2Cer	Ga2Cer 18:1;O2/17:0	875.6300	0.025	3	0.01
Sulfatides	SHexCer 18:1-D7;O2/13:0	761.5500	0.005	1.2	0.001
	SHexCer 18:1;O2/12:0	740.4860	0.005	1.35	0.001

**SPB**	**SPB 18:1-D7;O2**	306.3264	0.2	9	0.78
	**SPB 17:1;02**	285.2668	0.4	4.5	0.84

Table S6. Constructs used in this study.

Construct name	Amino acids
NF155 IgG1-Fn4 (FL ECD)	I25-G1043
NF155 IgG1-4	I25-S435
NF155 Fn1-4	R626-G1043
NF155 IgG5-Fn2	E528-D819
NF155 IgG5-Fn4	E528-G1043
NF155 IgG1-Fn2	I25-D819
NF186 IgG1-Fn4 (FL ECD)	I25-G1109

Supporting Methods

Lipidomics

For lipidomic analysis, 6 x 10^5 MO3.13 cells were seeded into 15 cm dishes and incubated for 24 hrs in DMEM containing 10% (v/v) FCS. Medium was then replaced with differentiation medium (as described above) and incubated for a further 7 days. Cells were washed twice in ice-cold PBS and scraped into microfuge tubes. Cells were gently centrifuged (500 *g*, 5 mins, 4°C) and residual PBS removed. Pellets were snap frozen in liquid nitrogen and stored at -80°C. Lipids were extracted according to the modified Folch protocol (6). Cell pellets (25–28 mg) were spiked with 10 µL of Internal Standard mixture (**Table S5**). Two mL of chloroform and 1 mL of MeOH were added. The resulting mixture was homogenised in an ultrasonic bath for 15 min at 40°C. When samples reached ambient temperature, 600 µL of 250 mM of ammonium carbonate in water was added, and the samples were homogenised in the ultrasonic bath at 40°C for 15 min, then centrifuged at 3000 rpm for 5 min, and the organic layer (bottom layer) was transferred using Pasteur pipettes to 8 mL glass vials. The extraction was repeated by adding 2 mL of chloroform to the aqueous phase. Then, the samples were sonicated in the ultrasonic bath at 40°C for 15 min, centrifuged at 3000 rpm for 5 min, and finally, the lower organic layer was transferred and combined with the first layer. The combined organic phases were evaporated under the nitrogen stream to dryness, and the extracts were stored at -80°C.

Prior to analysis, samples were dissolved in 250 µL of chloroform/MeOH (1:1, v/v) mixture. An Agilent 1290 Infinity series UHPLC system (Agilent Technologies, Waldbronn, Germany) was used for HILIC-UHPLC experiments with the following conditions: the column Acquity UPLC BEH HILIC (150 × 2.1 mm; 1.7 µm), the column temperature 40°C, the flow rate 0.25 mL/min, and the injection volume 1 µL. The mobile phase A was acetonitrile/water (96/4, v/v), the mobile phase B was acetonitrile/water (50/50, v/v) and both phases contained 15 mM of ammonium acetate. The following gradient elution was used: 0 min, 100 % A; 7.5 min, 84 % A; 11 min, 70 % A; 12 min, 100 % A; 22 min, 100 % A. The total run time including equilibration is 22 min. The autosampler temperature was set at 4°C. The UHPLC system was coupled to a Xevo G2-XS-QTOF mass spectrometer (Waters, Milford, MA, USA). The data were acquired in the sensitivity mode using ESI in the positive and negative ion mode using the following conditions: the capillary voltage of 3 kV for the positive ion mode and 1.5 kV for the negative ion mode, the sampling cone of 20 V, the source offset of 90 V, the source temperature of 150°C, the desolvation temperature of 500°C, the cone gas of 50 L/h, and the desolvation gas flow of 1000 L/h for both ion modes. Mass spectra were measured in the *m/z* range of 150–1500 with the scan time of 0.5 s using

the continuum mode and the lock mass scanning. The peptide leucine enkephalin was used as the lock mass for all MS experiments. MS/MS experiments were performed with a collision energy of 35 eV.

The measured data were submitted for noise reduction with the threshold of 20 by the Waters Compression Tool and the lock mass correction was subsequently applied to obtain accurate masses, and the data were converted to the centroid mode. The text files including summarized *m/z* with the corresponding intensities were exported by MarkerLynx XS software. The MS data were processed by a laboratory-made Excel macro script named LipidQuant with an embedded database (7). Lipid species were detected according to accurate *m/z* values with the mass tolerance of 10 mDa. The quantitative analysis was performed using internal standards, and isotopic correction II type was applied. The concentration is expressed in pmol/mg of cells.

The lipids with average concentration lower than 0.1 pmol/mg were excluded for statistical evaluation. Absolute concentrations of lipid species were used for MDA by SIMCA software. The Pareto scaling and logarithm transformation were performed for the data normalisation. Differences were investigated using statistical projection methods PCA, OPLS-DA, and S-plot (**Fig. S2A** and **C**). The quantification data were transformed using a base 2 logarithm. For fold change, the average of the $\log_2$ Cas9 control replicates was subtracted from the average of the $\log_2$ of the knockout replicates and plotted with standard errors (**Fig. S2B** and **D**).

Plasma Membrane Profiling (PMP) and Data Processing

PMP labelling and streptavidin enrichment. For PMP analysis, 6 x 10^5 MO3.13 cells were seeded into 15 cm dishes and incubated for 24 hrs in DMEM containing 10% (v/v) FCS. Medium was then replaced with differentiation medium (as described above) and incubated for a further 7 days. PMP was performed as described previously with minor modifications (8, 9). Cells were washed twice in ice-cold PBS. Surface sialic acid residues were oxidised and biotinylated by incubation in the dark for 30 min with gentle rocking at 4°C using an oxidation/biotinylation mix comprising 1 mM sodium meta-periodate, 100 µM aminooxy-biotin (Biotium Inc., Hayward, CA) and 10 µM aniline (Sigma-Aldrich) in ice-cold PBS, pH 6.7. The reaction was quenched with 1 mM glycerol and cells were washed twice in ice-cold PBS. Biotinylated cells were scraped into lysis buffer (10 mM Tris pH 7.6, 1% Triton X-100, 150 mM NaCl, protease inhibitor (complete, without EDTA (Roche), ½ tablet per 20 mL), and 5 mM EDTA) and incubated on a rotating wheel at 4°C for 30 mins. Lysate was cleared by centrifugation (20,000 *g*, 10 mins) at 4°C and supernatant snap frozen in liquid nitrogen and stored at -80°C until further processing. Protein concentrations of thawed samples were determined by bicinchoninic acid (BCA)

assay and equal quantities of samples used in subsequent steps. Biotinylated glycoproteins were enriched by incubation for 3 hrs at 4°C on a rotating wheel with high affinity streptavidin agarose beads (ThermoFisher Scientific). Extensive washing was performed on a vacuum manifold, using lysis buffer, then PBS containing 0.5% (w/v) SDS then washed with urea buffer (6 M urea, 50 mM Triethylammonium bicarbonate, TEAB). Beads were incubated at room temperature with shaking for 30 mins in reduction/alkylation mix (urea buffer containing 20 mM iodoacetamide and 10 mM TCEP). Beads were further washed with urea buffer and then 50 mM TEAB and transferred to a fresh tube. Beads were pelleted gently (550 *g*, 2 mins), supernatant removed. Captured protein was digested on-beads in 100 µL of 50 mM TEAB with 1 µg trypsin (Pierce, MS-grade) at 37°C with shaking for 4 hrs. Beads were pelleted gently and supernatant containing trypsinised peptides were dried using a speedvac (Thermo Scientific).

TMT Labelling and clean-up. Samples were resuspended in 21 µL 100mM TEAB pH 8.5. After allowing to come to room temperature, 0.5 µg TMTpro (GALC-KO) or 0.2 µg TMT reagents (UGT8-KO) (Thermo Fisher) were resuspended in 9 µL anhydrous acetonitrile (ACN) which was added to the respective samples and incubated at RT for 1 hr. For the GALC-KO experiment, TMTpro reagents were used in ascending order of reporter molecular mass to label: 3× WT, 3× Cas9 only, 3× GALC-KO G1, 3× GALC-KO G2 and 3× GALC rescue. For the UGT8-KO experiment, TMT reagents were similarly used to label: 3× Cas9 only, 3× UGT8-KO G1, 3× UGT8-KO G2. A 3 µL aliquot of each sample was taken and pooled to check TMT labelling efficiency and equality of loading by LC-MS. Samples were stored at -80°C in the interim. After checking each sample was at least 98% TMT labelled, total reporter ion intensities were used to normalise the pooling of the remaining samples such that the final pool should be as close to a 1:1 ratio of total peptide content between samples as possible. This final pool was then dried in a vacuum centrifuge. The sample was acidified to a final 0.1% Trifluoracetic Acid (TFA) (~200µL volume) and Formic Acid (FA) was added until the sodium deoxycholate (SDC) visibly precipitated. Four volumes of ethyl acetate were added and the sample vortexed vigorously for 30s. Sample was then centrifuged at 15,000 *g* for 5 mins at RT to effect phase separation. A gel loading pipette tip was used to withdraw the lower (aqueous) phase to a fresh low adhesion microfuge tube. If any obvious SDC contamination remained the two-phase extraction with ethyl acetate was repeated. The sample was then partially dried in a vacuum centrifuge and brought up to a final volume of 1mL with 0.1% TFA. FA was added until the pH was <2, confirmed by spotting onto pH paper. The sample was then cleaned up by solid phase extraction using a 50 mg tC18 SepPak cartridge (Waters) and a positive pressure manifold (Tecan Resolvex M10). The cartridge was wetted with 1 mL 100% methanol followed by 1 mL ACN, equilibrated with 1 mL 0.1% TFA and the sample loaded slowly. The cartridge

was washed with 1 mL 0.1% TFA before eluting with 750 µL 80% ACN, 0.1% TFA. Eluate was dried in a vacuum centrifuge.

Basic pH reversed phase fractionation. Samples were resuspended in 40 µL 200 mM ammonium formate pH 10 and transferred to a glass HPLC vial. High pH reverse phase fractionation was conducted on an Ultimate 3000 UHPLC system (Thermo Scientific) equipped with a 2.1 mm × 15 cm, 1.7 µm Kinetex EVO column (Phenomenex). Solvent A was 3% ACN, Solvent B was 100% ACN, solvent C was 200 mM ammonium formate pH 10. Throughout the analysis solvent C was kept at a constant 10%. The flow rate was 500 µL/min and UV was monitored at 280 nm. Samples were loaded in 90% A for 10 min before a gradient elution of 0–10% B over 10 min (curve 3), 10–34% B over 21 min (curve 5), 34–50% B over 5 min (curve 5) followed by a 10 min wash with 90% B. Fractions (100 µL) were collected throughout the run. Fractions containing peptide (as determined by A280) were recombined across the gradient to preserve orthogonality with on-line low pH RP separation. For example, fractions 1, 25, 49, 73, 97 are combined and dried in a vacuum centrifuge and stored at -20°C until LC-MS analysis. Twelve fractions were generated in this way.

Mass Spectrometry. Samples were analysed on an Orbitrap Fusion instrument on-line with an Ultimate 3000 RSLC nano UHPLC system (Thermo Fisher). Samples were resuspended in 10 µL 5% DMSO/1% TFA and loaded via a trapping column. Trapping solvent was 0.1% TFA, analytical solvent A was 0.1% FA, solvent B was ACN with 0.1% FA. Samples were loaded onto a trapping column (300 µm × 5mm PepMap cartridge trap; Thermo Fisher) at 10 µL/min for 5 minutes. Samples were then separated on a 75 cm × 75 µm i.d. 2 µm particle size PepMap C18 column (Thermo Fisher). The gradient was 3-10% B over 10 mins, 10-35% B over 155 minutes, 35-45% B over 9 minutes followed by a wash at 95% B for 5 mins and re-equilibration at 3% B. Eluted peptides were introduced by electrospray to the MS by applying 2.1 kV to a stainless steel emitter (5 cm x 30 µm; PepSep). During the gradient elution, mass spectra were acquired with the parameters detailed in **Fig. S16A** using Tune v3.3 and Xcalibur v4.3 (Thermo Fisher). For TMTpro/TMT experiments the isobaric loss exclusion settings were adjusted appropriately.

Data Processing. Data were processed with PeaksX+, v10.5 (Bioinfor). Processing parameters are shown in detail in **Fig. S16B**. Briefly, .raw files were searched iteratively in three rounds, with unmatched DeNovo spectra, at 0.1% peptide spectrum match (PSM) false discovery rate (FDR), from the previous search used as the input for the next. The three iterations were as follows: 1) Swissprot Human (27/03/2020) + common contaminants; 2) The same databases as search 1 but permitting semi-specific cleavage; 3) trEMBL Human (27/03/2020), with specific cleavage rules. Proteins were then quantified using the parameters outlined in **Fig. S16C**. Identified proteins and their TMT reporter

abundances were output to .csv, imported to R and submitted to statistical analysis using LIMMA, a moderated t-test available through the Bioconductor package (10). LIMMA *p*-values were corrected for multiple hypothesis testing using the Benjamini-Hochberg method to generate an FDR (*q*-value) for each comparison. The mass spectrometry proteomics data have been deposited to the ProteomeXchange Consortium via the PRIDE (11) partner repository with the dataset identifiers PXD036727 and 10.6019/PXD036727.

I3N neuronal cell culture

Human CRISPRi-i3N induced pluripotent stem cells (iPSCs; generated in a WTC11 iPSC background line) were a gift from Michael Ward (NIH). iPSCs were cultured in Essential 8 (E8) medium (STEMCELL Technologies) on dishes coated with Matrigel (Corning). E8 was replaced daily and cells were passaged at 80%–90% confluency with 0.5 mM EDTA to maintain colony growth and with the ROCK inhibitor Y-27632 (5 µM, Tocris). All cell lines were cultured with 5% CO2 at 37°C and were regularly tested for mycoplasma contamination. Differentiation into i3Neurons was as previously described, with slight modifications (12). Briefly, on day 0 iPSCs were dissociated into single cells using StemPro Accutase (Thermo Fisher Scientific) and seeded at a density of 8 x 10^5 cells/cm2 on Matrigel-coated culture dishes in Induction Medium (IM) composed of DMEM/F-12, 1× N-2 Supplement, 1× MEM Non-Essential Amino Acids Solution, 1× GlutaMAX Supplement (all Thermo Fisher Scientific), 10 µM Y-27632, and 2 µg/mL doxycycline hydrochloride (Sigma-Aldrich). Pre-differentiated cells were maintained in IM for 3 days with daily medium changes. After the 3-day differentiation period, cells were dissociated with StemPro Accutase and seeded at 1.5 × 10^5 cells/cm2 onto culture plates coated with 0.1 mg/mL poly-L-ornithine (Sigma-Aldrich). Cells were maintained in Cortical Neuron Culture Medium, composed of BrainPhys Neuronal Medium (STEMCELL Technologies), 1× B-27 Supplement (Thermo Fisher Scientific), 10 ng/mL BDNF (PeproTech), 10 ng/mL NT-3 (PeproTech), and 1 µg/mL mouse Laminin (Thermo Fisher Scientific) with half media changes carried out every 3–4 days.

CRISPRi gene silencing

Sense and antisense sgRNA oligonucleotides were designed with 5’CACC and 3’CAAA overhangs, respectively, and cloned into pKLV-U6gRNA-EF(BbsI)-PGKpuro2ABFP (BpiI) for lentivirus production. The following sgRNA were used: scrambled: GGGACGCGAAAGAAACCAGT; GALC KO1: AATGGCTGAGTGGCTACTCT; GALC KO2: CCGAGCGAAAGCTATGACTG, both GALC guides targeting exon 1. pKLV-U6gRNA-EF(BbsI)-PGKpuro2ABFP was a gift from Evan Reid, CIMR (Addgene plasmid # 50946;

RRID: Addgene_50946). For lentiviral production pKLV-hGALC was co-transfected into 5 x 10^5 HEK293-T cells with pMD VSV-G and pCMV Δ8.91 viral packaging plasmids at a ratio of 1:0.3:0.7 (gifts from Evan Reid, CIMR) using TransIT-293 transfection reagent. After 24 hrs, transfection medium was replaced with E8 medium (Gibco). 48 hours post transfection, virus-containing media was harvested, diluted 1:1 with fresh E8 medium, filtered through a 0.45uM filter and frozen at -80°C. Lentivirus was added to target cells and following spinoculation at 750 *g* for 1 h at 32°C, cells were incubated at 37°C overnight. On the following day, the media was changed for fresh 2ml E8 medium. On the subsequent day, selection was begun with fresh E8 + 1ug/ml puromycin for 48 hrs.

Quantitative PCR from i3N cell lines

I3N for qPCR were seeded into PLO coated 6 well dishes at a density of 1.5x10^6 cells/well and were harvested at 14 days of differentiation. RNA was extracted using a Purelink RNA extraction kit (Invitrogen) following the manufacturer's instructions. RNA was reverse transcribed to cDNA using a High-Capactiy RNA-to-cDNA kit (Thermo Fisher). qPCR experiments were conducted with technical and biological triplicates. DNA amplification was performed using TaqMan FAM labelled probes (Hs00164660_m1). 20ul reactions containing 1x TaqMan master mix, 1x TaqMan assay probe, 100ng cDNA and RNAse free water were set up in white-bottomed 96-well plates and assayed on a CFX96 real-time thermal cycler (Bio-Rad). The protocol included 40 cycles of melting at 95°C for 15 seconds, annealing and extension at 60°C for 1 minute, followed by a fluorescence measurement of all wells. Expression changes were measured using the ΔΔCt method. Statistical significance of fold changes were determined using the one-way anova statistical test in Graph-pad prism 9.

I3N Sample preparation for PMP-MS

Cell lines for PMP-MS analysis were plated in triplicate to yield three independent biological replicates. Partially differentiated D3 I3N lines of SCRM, GALC KO1 and KO2 cells were seeded at 12x10^6 cells/ plate in PLO coated 10cm plates and differentiated until their 14 day time-point with bi-weekly half media changes. All sample preparation for PMP-MS was done as descibed above.

Supporting References

1. Brinkman EK, Chen T, Amendola M, & van Steensel B (2014) Easy quantitative assessment of genome editing by sequence trace decomposition. *Nucleic Acids Res* 42:e168.

2. Waterhouse AM, Procter JB, Martin DM, Clamp M, & Barton GJ (2009) Jalview Version 2--a multiple sequence alignment editor and analysis workbench. *Bioinformatics* 25:1189-1191.

3. Scheres SH & Chen S (2012) Prevention of overfitting in cryo-EM structure determination. *Nat Methods* 9:853-854.

4. Pettersen EF, Goddard TD, Huang CC, Meng EC, Couch GS, Croll TI, Morris JH, & Ferrin TE (2021) UCSF ChimeraX: Structure visualization for researchers, educators, and developers. *Protein Sci* 30:70-82.

5. Croll TI (2018) ISOLDE: a physically realistic environment for model building into low-resolution electron-density maps. *Acta Crystallogr D Struct Biol* 74:519-530.

6. Wolrab D, Chocholouskova M, Jirasko R, Peterka O, Muzakova V, Studentova H, Melichar B, & Holcapek M (2020) Determination of one year stability of lipid plasma profile and comparison of blood collection tubes using UHPSFC/MS and HILIC-UHPLC/MS. *Anal Chim Acta* 1137:74-84.

7. Wolrab D, Cifkova E, Can P, Lisa M, Peterka O, Chocholouskova M, Jirasko R, & Holcapek M (2021) LipidQuant 1.0: automated data processing in lipid class separation-mass spectrometry quantitative workflows. *Bioinformatics* 37:4591-4592.

8. Weekes MP, Antrobus R, Lill JR, Duncan LM, Hor S, & Lehner PJ (2010) Comparative analysis of techniques to purify plasma membrane proteins. *J Biomol Tech* 21:108-115.

9. Weekes MP, Tomasec P, Huttlin EL, Fielding CA, Nusinow D, Stanton RJ, Wang ECY, Aicheler R, Murrell I, Wilkinson GWG, Lehner PJ, & Gygi SP (2014) Quantitative temporal viromics: an approach to investigate host-pathogen interaction. *Cell* 157:1460-1472.

10. Ritchie ME, Phipson B, Wu D, Hu Y, Law CW, Shi W, & Smyth GK (2015) limma powers differential expression analyses for RNA-sequencing and microarray studies. *Nucleic Acids Res* 43:e47.

11. Perez-Riverol Y, Bai J, Bandla C, Garcia-Seisdedos D, Hewapathirana S, Kamatchinathan S, Kundu DJ, Prakash A, Frericks-Zipper A, Eisenacher M, Walzer M, Wang S, Brazma A, &

Vizcaino JA (2022) The PRIDE database resources in 2022: a hub for mass spectrometry-based proteomics evidences. *Nucleic Acids Res* 50:D543-D552.

12. Fernandopulle MS, Prestil R, Grunseich C, Wang C, Gan L, & Ward ME (2018) Transcription Factor-Mediated Differentiation of Human iPSCs into Neurons. *Curr Protoc Cell Biol* 79:e51.
